# WHIRLY1 Interactome Profiling Reveals the Chloroplast Fate Correlation with Stromule-like Morphologies in *Arabidopsis* Leaves Under Dark and DBMIB Stress Conditions

**DOI:** 10.3390/plants15192930

**Published:** 2026-09-24

**Authors:** Mengsi Li, Dongmei Huang, Youwei Yu, Kanghui Zhang, Ping Yang, Yuhao Qiu, Xiangzi Zheng, Wenfang Lin, Yujun Ren, Binghua Wu, Ying Miao

**Affiliations:** 1 Fujian Provincial Key Laboratory of Plant Functional Biology, Fujian Agriculture and Forestry University, Fuzhou 350002, China; limengsi3391@126.com (M.L.); 12405014037@fafu.edu.cn (Y.Y.); hwzdpt@163.com (K.Z.); yangping1109@163.com (P.Y.); qiuyh35@mail2.sysu.edu.cn (Y.Q.); zxiangzi8353@126.com (X.Z.); linwf@fafu.edu.cn (W.L.); ryj@fafu.edu.cn (Y.R.); binghua.wu@fafu.edu.cn (B.W.); 2 Department of Biochemistry and Molecular Biology, Xiamen Medical College, Xiamen 361023, China; hdm_1112@126.com

**Keywords:** stromule-like morphology, chloroplast function, interactome, stresses

## Abstract

The WHIRLY family comprises DNA/RNA-binding proteins localized to multiple subcellular compartments, including organelles and the nucleus. However, the subcellular distribution and functional roles of WHIRLY protein complexes are not fully characterized. In this study, a WHIRLY1 protein pulldown coupled with mass spectrometry analysis was used to profile the interacting proteome in an *Arabidopsis thaliana* plant overexpressing WHIRLY1–GFP. This analysis identified over 770 interacting proteins, with RNA-binding proteins representing the most enriched functional category. Subcellular compartmental mapping revealed distinct spatial organization: oxidation-related, cytoskeleton-associated, and chloroplast/mitochondrion-targeted RNA-binding proteins were significantly enriched at the chloroplast periphery. Conversely, a subset of nuclear envelope-associated proteins, including NOT3, TUBB3, and histone H4, were connected with the formation of tubular stromule-like structures under growth light conditions as opposed to extended dark conditions. Under DBMIB-induced stress, however, reconfiguration of the WHIRLY1 interactome occurred: plastid ribosomal structural components declined, while the transcription factor GRF6 accumulated in the nucleus, concomitant with the formation of vesicular stromule-like structures. Biomolecular fluorescence complementation and yeast two-hybrid assays confirmed direct interactions between WHIRLY1 and stromule-localized proteins, several RNA-binding proteins, and nuclear proteins. Collectively, these findings suggest that the dynamic assembly of the WHIRLY1 interactome in conjunction with stromule biogenesis acts as a regulatory nexus coordinating chloroplast functions under stressful conditions.

## 1. Introduction

Plants have evolved sophisticated, organelle-level mechanisms to maintain cellular homeostasis and defend against biotic and abiotic stresses. Inter-organellar communication serves as a fundamental regulatory strategy that translates diverse environmental stimuli into coordinated cellular acclimation responses, primarily through dynamic modulation of metabolic and catabolic activities to sustain optimal growth under fluctuating conditions [1].

Chloroplast morphology exhibits pronounced plasticity in response to environmental stress, facilitating the establishment of multiple inter-organellar contact sites. Well-documented examples include plastid–nucleus complexes, plastid-derived vesicles, and stromules (stroma-filled tubular plastid extensions) [2,3,4]. Plastid–nucleus complexes enable direct physical apposition between plastids and the nucleus, thereby facilitating the targeted transfer of photosynthesis-derived signaling molecules, including hydrogen peroxide (H_2_O_2_) and small metabolites, to the nucleus under high-light conditions [5,6,7]. Plastid-derived vesicles are implicated in the selective removal of misfolded proteins and damaged chloroplast components, such as stromal, envelope, and thylakoid proteins, via membrane-bound transport to the vacuole for degradation [3]. Stromules extend from chloroplasts and dynamically associate with the nucleus, endoplasmic reticulum (ER), and plasma membrane (PM) in *Arabidopsis thaliana* [8,9]. Their extension and retraction are tightly regulated by abiotic and biotic cues, including high light, heat, reactive oxygen species (ROS), phytohormones, sugars, and pathogen-associated molecular patterns (PAMPs), suggesting a central role in mediating inter-organellar signaling [10]. Indeed, stromules are proposed to function as conduits for the bidirectional trafficking of signaling molecules, including metabolites and proteins, between plastids and other organelles [1,11,12,13,14,15,16].

An increasing amount of evidence identifies several proteins essential for stromule formation and function: the plastid inner envelope protein LACS9 [17]; the outer-envelope protein OEP7 and the transmembrane-domain CHUP1 protein [18]; and the actin-binding protein KIS1 [19]. Notably, WHIRLY1 (WHY1) has been shown to interact with the light-harvesting complex protein LHCA1 under illumination, inducing the formation of prominent stromule-like structures [20]. However, the molecular mechanism governing WHY1’s nucleus–cytoplasmic shuttling and its potential relocalization to plastids remains incompletely understood.

WHIRLY (WHY) proteins constitute a conserved family of single-stranded DNA/RNA-binding proteins. Among the three *Arabidopsis* WHY proteins, WHY1 and WHY3 exhibit dual localization to chloroplasts and the nucleus, whereas WHY2 displays triple localization to mitochondria, chloroplasts, and the nucleus [21,22,23,24,25]. WHY1 is the most extensively characterized isoform and functions in a context-dependent manner across subcellular compartments: (1) in the nucleus, WHY1 acts as a transcriptional regulator modulating the expression of genes involved in leaf senescence and stress adaptation [26,27,28,29,30]; (2) within chloroplasts, WHY1 associates with transcriptionally active nucleoids, where it binds DNA and RNA and interacts with redox regulators such as thioredoxin z (TRXz), contributing to chloroplast RNA metabolism and processing [31,32,33]; (3) as an anti-recombinogenic factor, WHY1 interacts with recombinase proteins (e.g., RECA and RDHs) in organelles to ensure the faithful repair of DNA double-strand breaks (DSBs), thereby safeguarding organellar genome integrity [34,35,36]; and (4) it also localizes to the endosomal membranes of plastids, where its interaction with LHCA1 implicates it in redox-, nitrogen-, temperature-, and hormone-responsive signaling pathways [20,37,38,39,40,41]. Although previous studies have documented the stimulus-induced redistribution of WHY1 between chloroplasts and the nucleus upon treatment with abscisic acid (ABA), H_2_O_2_, or salicylic acid (SA) [39,40,42], the composition, regulation, and functional dynamics of the WHY1-containing protein complexes remain poorly defined.

In this study, we used a stable transgenic *Arabidopsis* line that overexpresses WHY1–GFP to induce the formation of stromule-like structures under controlled conditions, including exposure to light and darkness and treatment with the photosynthetic electron transport inhibitor DBMIB. Comparative WHY1 affinity purification, coupled with quantitative mass spectrometry, enabled the systematic identification of WHY1-interacting proteins. Integrative analysis of differentially enriched proteins, including nuclear and plastid isoform-specific accumulated proteins, was subsequently performed to elucidate the context-dependent assembly and functional specialization of the WHY1 interactome. Results from a bimolecular fluorescence complementation assay and a yeast two-hybrid assay suggested that the WHY1 complex, with the alteration of stromule-like structure formation, operates as a highly integrated platform that is involved in multiple steps of chloroplast function.

## 2. Results

### 2.1. Comparative Proteomic Profiling of the WHY1–GFP Interactome Associated with Stromule-like Structural Remodeling

Previous studies have established WHY1 as a stress-responsive, dual-localized protein that orchestrates diverse molecular functions in both the nucleus and chloroplasts through context-specific protein–protein interactions [41,43]. To comprehensively characterize the composition and condition-dependent dynamics of the WHY1 protein complex, we employed a stable transgenic *Arabidopsis thaliana* line expressing WHY1–GFP under its native promoter. Six-week-old rosette leaves were subjected to three defined normal growth light conditions: (i) extended darkness (9 h), (ii) normal photoperiodic light (control), and (iii) normal light conditions treated with 12 µM DBMIB (a specific inhibitor of the cytochrome b_6_f complex that blocks photosynthetic electron transport at photosystem II), for two hours [14,44]. As expected, WHY1 protein abundance decreased significantly under darkness treatment and increased significantly under DBMIB treatment relative to the normal light condition (Figure S1A–B), confirming its responsiveness to redox and energetic cues. To decouple changes in WHY1 abundance from alterations in complex composition, we utilized WHY1–GFP overexpression lines to ensure consistent bait expression across conditions and performed GFP-trap affinity purification followed by quantitative mass spectrometry. Immunoblotting and RT–qPCR confirmed stable WHY1 transcript and protein levels across all treatments (Figure 1A,B), thereby validating experimental comparability. Consistent with previous reports (manuscript, in revision), the confocal imaging and electron microscopy observations revealed distinct plastid morphologies: direct plastid–nucleus membrane apposition under extended darkness (Figure 1C(a), Figure 1D(a), respectively); abundant tubular stromules under growth light conditions (Figure 1C(b), Figure 1D(b), respectively); and numerous vesicle-like stromule derivatives upon DBMIB treatment (Figure 1C(c), Figure 1D(c), respectively).

Total soluble proteins extracted from each treatment group were subjected to GFP-based immunoprecipitation (IP) using GFP-binding protein (GBP) resins. Parallel control experiments included non-induced *oeGFP* and *oeWHY1–GFP* plants maintained under standard light conditions to define background binding and identify WHY1-specific interactors (Figure 1E). Initial validation demonstrated robust enrichment: 1775 proteins were significantly enriched in WHY1–GFP IP samples relative to GFP-only controls (Figure S2A), confirming assay sensitivity and specificity. All subsequent comparative analyses were conducted using ≥3 independent biological replicates per condition (Figure S2B). Principal component analysis (PCA) of the quantitative proteomics dataset revealed clear separation among treatment groups, with one light replicate exhibiting minor deviation consistent with inherent biological variability but not compromising overall statistical power (Figure 1F).

For functional interpretation, we applied Gene Ontology (GO) and pathway enrichment analysis using Fisher’s exact test, with the entire quantified GFP-only IP proteome serving as the background reference. Multiple-testing correction was performed using the Benjamini–Hochberg procedure (false discovery rate, FDR < 0.05); only categories meeting this stringent threshold were deemed statistically significant. This yielded 1078 differentially enriched proteins (DEPs) in the dark versus light comparison (D/L), and 896 DEPs in the L-DBMIB versus light comparison (L-DBMIB/L) (Figure 1G; Dataset S1). Overlap analysis identified 777 DEPs common to both comparisons (Figure 1G). Further stratification revealed 99 DEPs that were significantly enriched in light relative to dark (L/D), comprising 41 proteins uniquely detected in light and 58 uniquely detected in dark; 137 proteins significantly enriched in DBMIB relative to dark (L-DBMIB/D); and 24 proteins significantly enriched in DBMIB relative to light (L-DBMIB/L) (Figure 1H; Dataset S1). Collectively, these data suggest that under normal growth light conditions, 99 WHY1-associated proteins might be involved in tubular stromule formation, whereas a pronounced reduction in the number of 137 WHY1-associated proteins might be associated with DBMIB-induced vesicular stromule formation. This suggests that inhibition of photosynthetic electron transport triggers the selective disassembly or displacement of a significant portion of the WHY1 interactome.

### 2.2. RNA-Binding Proteins and Cytoskeletal Proteins Were Significantly Enriched in the WHY1-Associated Protein Complex Under Normal Growth Conditions

During WHY1 immunoprecipitation-based proteomic profiling, we identified robust enrichment of WHY1 with canonical stromal proteins (Gene Ontology term GO:0010319), including NRIP1 [11,12], allene oxide cyclase (AOC2; At3g25770), chloroplast stem–loop binding protein CSP41a [45] (At3g63140), malate dehydrogenase (AT3G47520), fructose-bisphosphatase-associated high cyclic electron flow protein 1 (HCEF1), assimilatory sulfite reductase (ferredoxin-dependent; AT3G54050), glyceraldehyde-3-phosphate dehydrogenase A (GAPA), NAD-binding proteins [46], RuBisCO activase (RCA), and NAD(*p*)-dependent oxidoreductases. Additionally, membrane organization factors, including thylakoid formation factor VIPP1 and SNARE complex subunits, as well as stress-responsive proteins, such as cold-shock protein CSP41a and FIBRILLIN, were consistently co-immunoprecipitated with WHY1 (Table 1; Table S1; Dataset S1). Collectively, these findings suggested that the WHY1 interactome is functionally and spatially associated with stromule-like structures in *Arabidopsis* under growth light conditions.

Gene ontology (GO) analysis of the 1078 differentially expressed proteins (DEPs) revealed pronounced subcellular compartmentalization: significant enrichment (*p* < 0.01) was observed in biological processes linked to environmental response; molecular functions encompassing RNA binding (q = 1.2 × 10^−6^) and carboxy-lyase activity (q = 7.8 × 10^−5^); and cellular components localized to plastids, mitochondria, and cytoskeletal networks (Figure 2A). Among the light-induced DEPs, 28 of 78 RNA-binding proteins (35.9%) and 14 cytoskeletal regulators, including α-tubulin isoforms (TUA1/TUA6), β-tubulin isoforms (TUB3/TUB8/TUB9), and actin-binding proteins ACT1 and VLN2, showed increased abundance (Figure 2B; Dataset S2). This coordinated accumulation correlates with light-triggered formation of WHY1-associated stromule-like structures.

Compartment-specific enrichments further delineated functional modules: (i) peroxisomal enzymes, including catalase 2/3 (CAT2/CAT3) and FMN-dependent oxidoreductases; (ii) plastid–mitochondrial interface proteins, such as RuBisCO large subunit (rbcL) and CURT1B, a thylakoid architecture regulator; (iii) vesicle trafficking machinery, including SNARE complex components and lipid-transfer protein LOX2; and (iv) nucleocytoplasmic shuttling factors, such as RNA helicase RH11 and GRF6, a phosphor-sensor implicated in signal transduction. Biological process mapping additionally uncovered stress-responsive regulators, including RD29A and LEA proteins, and developmental modulators, including GRP3 and TPR-domain-containing proteins, within the light-induced WHY1-associated proteome (Figure 2B). Together, these data hint at a model wherein WHY1 suggests the spatiotemporal coordination of translational regulation (via recruitment of RNA-binding proteins), redox homeostasis (through peroxisomal and plastidial antioxidant networks), and cytoskeletal dynamics under growth light conditions distinct from dark-extended states.

### 2.3. Ribosomal Structural Proteins Were Significantly Depleted in the WHY1-Associated Protein Complex Under DBMIB-Induced Stress Conditions

In the WHY1 immunoprecipitation-based proteomic comparison of DBMIB-treated versus light-grown samples (IP-L-DBMIB vs. IP-L), 896 differentially expressed proteins (DEPs) were identified. Stromule-associated and cytoskeletal proteins were consistently enriched in the IP-L-DBMIB interactome relative to IP-L (Figure 2C). In contrast, DBMIB treatment triggered pronounced ribosomal disassembly: 137 of 246 ribosomal proteins (55.7%) exhibited statistically significant depletion (*p* < 0.05; peptide-spectrum matches ≥ 2). Affected components spanned multiple functional tiers of the translation apparatus, including nine of the thirteen subunits of the 48S preinitiation complex, core proteins of both small (e.g., PSRP3 and PSRP5) and large ribosomal subunits, and key translation elongation factors (Figure 2D; Table S2). These ribosomal proteins’ decline was evidenced by log_2_ fold-changes ranging from −2.1 to −5.8 and was associated with the systemic downregulation of macromolecular biosynthesis (GO:0043170; *p* = 4.5 × 10^−9^) and nitrogen assimilation (GO:1901566; *p* = 2.1 × 10^−6^). The reciprocal regulation between DBMIB stress-induced vesicular stromules and ribosomal abundance indicates that the WHY1-associated vesicular stromule-like structure might function as a spatial regulatory hub that modulates translational capacity via compartmentalized sequestration of ribosomal and translation-related machinery.

### 2.4. A Spatially Resolved Map of the WHY1-Associated Protein Network Bridging Chloroplasts and the Nuclear Envelope

To systematically delineate the compartment-specific architecture of the WHY1 interactome, we leveraged complementary genetic tools: transgenic *Arabidopsis* lines expressing nuclear-localized WHY1–GFP (*OE-nWHY1–GFP/why1*) or plastid-localized WHY1–GFP (*OE-pWHY1–GFP/why1*) in the *why1* null background [40], alongside oeGFP-only and chloroplast transit signal peptide-fused GFP (oeCTP-GFP) controls (Figure S3A,B). Affinity purification coupled with mass spectrometry (AP-MS), using GFP nanobody-conjugated beads, identified 151 putative nuclear interactors and 426 putative plastid-localized interactors of WHY1 (Figure S3C; Dataset S4). Integrative analysis of these spatially segregated proteomes enabled the reconstruction of proximal, compartment-resolved WHY1 protein interaction networks.

Comparative interactome profiling revealed stress condition- and localization-dependent enrichment patterns. Overlapping analysis of the DEPs of growth light vs. extended dark (L/D) conditions, with 151 putative nuclear interactors and 426 putative plastid-localized interactors, respectively, revealed that the WHY1 interactome comprised 50 chloroplast-localized proteins, three nuclear proteins (TUB3, NOT3, and HISTONE H4), and 1025 cytoplasmic/membrane-associated proteins (Figure 3A,B). Overlapping analysis of the DEPs of growth light vs. L-DBMIB-treated (L/L-DBMIB) conditions, with 151 putative nuclear interactors and 426 putative plastid-localized interactors, respectively, revealed that it comprised 41 chloroplast proteins (34 overlapping with L/D), one nuclear protein (GRF6), and 734 cytoplasmic/membrane-associated proteins (Figure 3A,B). Strikingly, 15 chloroplast proteins, including CURT1B, the PSII 10 kDa protein, LHCA1, and RACK1A, were specifically induced under light and overlapped with canonical stromal proteins (Gene Ontology term GO:0010319); conversely, 34 chloroplast proteins showed coordinated downregulation under DBMIB treatment and were associated with vesicular stromule-like morphologies (Figure 3C,D). Functional annotation of the integrated interactome uncovered three major functional clusters: (i) photosynthetic regulation, encompassing 12 proteins enriched in carbohydrate metabolism (GO:0005975); (ii) translation machinery, including 22 ribosomal proteins (e.g., RPS8 and RPL2) and protein quality control factors; and (iii) cytoskeletal coordination, featuring ACT7, FtsZ, and VLN2, which physically associate with RNA-processing regulators such as CSP41a and TSN2 (Figure 3C). Notably, light-dependent accumulation of TUB3 (a structural GTPase), NOT3 (an RNA stability regulator), and histone H4 was observed specifically at the nuclear envelope within the nuclear isoform (nWHY1) complex (Figure 3C). In contrast, DBMIB treatment seemed to selectively recruit the 14-3-3 protein GRF6 to the nuclear WHY1 complex (Figure 3C). GRF6 is a well-characterized stress-responsive scaffold known to regulate autophagy initiation and nucleocytoplasmic trafficking [59,60]. Collectively, these spatiotemporally resolved recruitment patterns suggested that WHY1 nucleates functionally distinct structures associated with different kinds of stromule-like structures that coordinate inter-organellar communication, particularly between plastids and the nucleus.

### 2.5. WHY1 Complex Assembly Was Confirmed Across Multiple Subcellular Compartments Using Bimolecular Fluorescence Complementation (BiFC)

To extend the functional characterization of the WHY1-associated stromule complex, we first confirmed the interaction of WHY1 with a chloroplast-derived defense protein, NRIP1, which exists at tubular stromules [11,12], using the Venus-BiFC assay and co-immunoprecipitation (CoIP). BiFC assays in *Arabidopsis* protoplasts revealed robust Venus fluorescence upon co-expression of WHY1–Vn (Venus N-terminal fragment) with candidate protein–Vc (Venus C-terminal fragment) constructs, confirming direct interaction and localization (Figure 4A). The results revealed that WHY1 interacted with NRIP1 through the WHIRLY domain region in chloroplasts (Figure 4B). The CoIP results showed that the NRIP1–HA protein could be detected in IP with an antibody against HA (Figure 4C), indicating that WHY1 is able to interact with the NRIP1 protein to form a complex in chloroplasts. Next, we systematically identified WHY1-associated proteins, particularly those implicated in ribosome biogenesis, post-transcriptional regulation, pre-mRNA splicing, and translational control. Notably, the results revealed that the Venus signals were detected in chloroplasts of tobacco protoplasts co-tranformed with WHY1–Vc and the candidate–Vn of chloroplast-localized interactors, including MORF2, CMS1, and TRXz1 (Figure 4D). In contrast, Venus signals detected in the nucleus of *Arabidopsis* protoplasts co-transformed with WHY1–Vc and the candidate–Vn of nuclear interactors, comprising GRF6, TUB3, NOT3, and HDA15, were used as a positive control based on prior validation by Huang et al. (2022) [61]. No interaction signal was detected between WHY1 and TUBB9 (Figure 4E). Crucially, co-expression of negative controls with WHY1–Vn and empty Vn or Vc vectors yielded no detectable fluorescence, substantiating the specificity of WHY1 interactions with MORF2, CMS1, and TRXz1 in chloroplasts, and with GRF6, TUB3, and NOT3 in nuclei. Independent validation via yeast two-hybrid (Y2H) assays further corroborated the interactions between WHY1 and MORF2, CMS1, TRXz, GRF6, TUB3, and NOT3 in yeast cells (Figure 4F). Collectively, these results suggest that WHY1 acts as a central hub in chloroplasts and the nucleus, and that it is involved in the coordination of diverse molecular processes, including ribosomal assembly, RNA metabolism, and translational regulation.

Notably, among the 777 overlapping DEPs under both darkness and DBMIB stress conditions (relative to the normal growth light condition identified in the WHY1 interactome), 136 were annotated as components of non-membrane-bounded organelles (Table S3; Dataset S3). Strikingly, 36.6% of these proteins were predicted to contain intrinsically disordered regions (IDRs), apparently exceeding the genome-wide average of 25.9% (assessed using the Database of Disordered Protein Predictions, D2P2; https://d2p2.pro/, accessed on 25 July 2024; Figure 5A). This enrichment suggests that the dynamic reorganization of WHY1 complexes, particularly compared with the DBMIB-induced stress condition, might exist as an LLPS phenomenon. Furthermore, cross-referencing the 777 WHY1-associated proteins against a curated phase separation database [62]; a total of 985 LLPS-related proteins (Dataset S4) yielded 25 overlapped candidates (Figure 5B), encompassing (i) ribosomal proteins (e.g., RPS17, RPS28, RPL31, RPL33, and mRPL) previously linked to phase-separated ribonucleoprotein granules; (ii) cytoskeletal regulators (TUBA1, TUB3, TUBB9, and tubulin/FtsZ); (iii) RNA-processing factors (RRM-domain proteins, TUDOR2/TSN2, and pTAC6); (iv) metabolic enzymes (rbcL, GDCST, aminomethyltransferase, and catalase); and (v) structural regulators (DNA gyrase subunit B, RH11, CURT1B, and proteasome subunit beta). Interestingly, WHY1 and WHY3 were consistently identified among these candidates under both normal growth light conditions and DBMIB-treated conditions (Figure 5B,C).

Computational prediction using the PONDR algorithm (http://pondr.com/, accessed on 20 August 2023) indicated substantial intrinsic disorder regions in both WHY1 and WHY3 proteins, with disorder scores > 0.45 across multiple domains (Figure 5E,F). Live-cell imaging further showed that WHY1 spontaneously forms dynamic, spherical condensates within chloroplasts (Figure 5G), a process fully abolished by DBMIB treatment. Fluorescence recovery after photobleaching (FRAP) quantification revealed that rapid fluorescence recovery was significantly higher in dark and light conditions than under DBMIB-treated conditions (t_1_/_2_ < 12 s; Figure 5H), which seems to be consistent with liquid-like condensate behavior mediated by continuous molecular exchange. Together, these preliminary biophysical and computational data suggest an LLPS-driven phenomenon potentially associated with the assembly and regulation of WHY1-containing macromolecular complexes under normal growth and stress-related conditions.

### 2.6. Loss of WHY1 and WHY3 Impaired Ribosomal Proteins and Photosynthetic Components

To gain mechanistic insight into WHY1’s role in plastid homeostasis, we reanalyzed our previously published quantitative proteomic dataset comparing the *why1why3* double mutant and wild-type (WT) *Arabidopsis* [63]. Gene Ontology (GO) enrichment analysis revealed that upregulated differentially expressed proteins (DEPs) in the *why1why3* mutant were significantly enriched in reactive oxygen species (ROS) metabolism, proteolysis (e.g., CLP3 proteases), and thylakoid biogenesis, highlighting proteins such as RHODase and TRXz (Table 2). Conversely, downregulated DEPs included VIPP1, PSRP3, PSRP5, core photosynthetic components (e.g., RuBisCO subunits), ribosomal recycling factor, and signal transduction regulators (Table 2, Figure S4), collectively indicating broad impairment in chloroplast protein synthesis, membrane organization, and redox regulation.

## 3. Discussion

Although stromules were first described over 60 years ago [64] and are induced during mycorrhizal symbiosis with plants and starch granule formation, as well as in response to sucrose or glucose application and abiotic and biotic stresses [65], the biological function and composition of stromules remain elusive. This study provides a spatially resolved interaction map of the WHY1-centered protein network that connects chloroplasts and the nuclear envelope through stromule formation in *Arabidopsis*. Under normal conditions, WHY1 interactome profiling demonstrates that the WHY1-associated protein complex is involved in the organization of a multifunctional complex with tubular stromules to coordinate RNA metabolism, translation, and protein quality control. Under oxidative stress conditions, the WHY1-associated protein complex connects with vesicle-like stromules that facilitate chloroplast remodeling and selective autophagy (Figure 6). Although the GFP-nanotrap combined with quantitative mass spectrometry has certain limitations, it has been widely applied in numerous studies [66,67,68,69]. We have further validated the protein–protein interactions. Therefore, the results are reliable. Currently, there is no stromule marker that can be used to detect stromule structures. We confirmed the interactions between WHY1 and a chloroplast-derived defense protein, NRIP1, which is present at tubular stromules [11,12]. However, we were unable to observe the colocalization of WHY1 and NRIP1 in stromules in protoplasts under normal growth conditions. Additional stress conditions may be required to induce stromule formation in future research using a stable co-transformed WHY1–GFP/NRIP1–RFP transgenic plant.

Under normal growth light conditions, the well-characterized RNA-binding protein WHY1 [68] forms a spatially organized interactome associated with plastid tubular stromules. Our quantitative proteomic profiling significantly expands this interactome, including not only stromule-associated proteins and cytoskeletal regulators but also previously uncharacterized ribosomal components and their assembly factors. Comprehensive analysis suggests that the WHY1-associated stromule functions as an integrated regulatory platform that orchestrates three interdependent layers of gene expression: (1) RNA processing, including the core plastid transcription-active chromosome complex component pTAC6 [70], MORF2 and PPR proteins (essential for RNA editing and splicing in plastids [71,72]), and the CCR4-NOT2/3/5 subunits (multifunctional regulators of mRNA deadenylation, stability, and translational repression [73,74,75,76,77]); (2) translation regulation, exemplified by the plastid-specific ribosomal proteins PSRP3 and PSRP5 [78,79]; and (3) protein quality control, mediated by CLP proteases (which govern matrix pre-RNA processing, ribosome assembly, and the turnover of misfolded proteins [80]). Together, these findings support the long-standing ‘RNA and protein gating’ model for stromules [81] and suggest that WHY1 acts as a coordinator, spatially linking RNA binding, processing, translation, and proteostasis within the chloroplast–stromule–cytosol interface (Figure 6). Therefore, WHY1-associated stromule formation is not merely a passive conduit for protein trafficking, but rather an active, RNA-centric regulatory hub that is essential for maintaining the functional integrity of the chloroplast during vegetative growth. Importantly, our WHY1 affinity purification–mass spectrometry (AP-MS) approach captures the WHY1-bound stromule proteome. Non-interacting or transiently associated proteins were not enriched and thus lie outside the scope of this analysis.

Under DBMIB-induced oxidative stress, the WHY1-associated protein complex undergoes a functional reconfiguration: it appears to redistribute into vesicle-like stromules that accumulate around nuclei and chloroplasts. At the same time, the WHY1-associated proteome shows a significant reduction in ribosomal structural components (RPLs and RPSs) and translation initiation factors (e.g., eIF3 subunits), which suggests the inhibition of de novo chloroplast protein synthesis. This coincides with the upregulation of autophagy-related genes and the accumulation of unfolded/misfolded proteins, which is consistent with the activation of chloroplast-selective autophagy (chloroautophagy). Three non-exclusive mechanisms explain this stress-adaptive reorganization: (1) vesicle-like stromules serve as platforms for cargo sequestration and delivery to the autophagic machinery. GRF6, a WHY1 interactor localized to the nuclear envelope, has been shown to modulate ATG13 expression and autophagosome formation [59,60]. Secondly, stress has the potential to disrupt liquid–liquid phase separation (LLPS) signatures in the WHY1 interactome, as evidenced by the loss of IDR-enriched condensates (Figure 5). This suggests the dissolution of functional biomolecular condensates and a transition to an organelle-dysfunction state [82,83]. (3) Genetic loss of WHY1/WHY3 recapitulates key features of stress-induced dysfunction: downregulation of the ribosomal recycling factors PRSP3 and PRSP5, the vesicle-inducing plastid protein VIPP1 [84], and photosynthetic complexes, alongside upregulation of ROS-scavenging enzymes (e.g., RHODase and TRXz) [85] and proteolytic systems (e.g., the CLP3 protease) [86,87]. These molecular phenotypes align with the proteomic shifts observed under DBMIB stress, confirming that WHY1–stromule dynamics constitute a central regulatory axis governing chloroplast resilience versus collapse. However, these suppressed proteins excluded some of the increased proteins in growth conditions involving light relative to dark treatment (L/D), due to limitations in setting up a DBMIB treatment under extended dark conditions.

Chloroplasts and the nucleus engage in bidirectional signaling, termed anterograde (nucleus-to-organelle) and retrograde (organelle-to-nucleus) signaling, to dynamically coordinate nuclear gene expression in response to developmental cues and environmental stimuli [88,89,90]. This reciprocal communication is essential for integrating plastid status into the broader regulatory networks that govern plant growth, development, stress acclimation, and resource allocation in an evolutionarily dependent manner. In support of this paradigm, the Dinesh–Kumar group demonstrated that stromules extensively associate with nuclei during pathogen defense, coinciding with nuclear accumulation of the chloroplast-derived defense protein NRIP1 and hydrogen peroxide (H_2_O_2_). This implicates stromules in the amplification and targeted delivery of pro-defense signals to the nucleus and other subcellular compartments in *Arabidopsis* [11,12] and tobacco [2,14].

The WHIRLY protein family is evolutionarily conserved, although its sequence has variable regions at the N-terminus, which may differ across species. The conserved region of the WHIRLY domain exists in almost all plant species and functions as an RNA/ssDNA-binding region. This has been well documented by Krupinksa et al. (2022) [43], Muti et al. (2024) [91], and Lin et al. (2024) [41]. The WHIRLY domain also appears to be a protein–protein interaction site (Figure 5B). Foyer et al. (2014) [38] and Lin et al. (2024) [41] hypothesized the potential functions of WHY1 in stromules based on experimental observations of stromule structures related to WHY1 in barley, tobacco, rice, and Arabidopsis [20,43] (unpublished data for tobacco and rice in our laboratory). Based on these findings, our study identified multiple nuclear proteins, including α-tubulin 3 (TUB3), GRF6 (a nuclear membrane protein), NOT3 (a CCR4-NOT transcriptional regulator), and H4 (a core histone), within the stromule-associated proteome under normal growth conditions and DBMIB-induced stress conditions. Notably, we also detected the histone deacetylase HDA15, which is a previously validated nuclear interactor of WHY1, whose association with WHY1 exhibits developmental stage dependency in Arabidopsis [41,61,92]. Furthermore, this is consistent with the established interactions of WHY1 with the RNA-editing factors MORFs and the thioredoxin TRXz in rice [33,41]. Here, we observed co-enrichment of MORF2, CMS1, TRXz, H4, GRF6, NOT3, and TUB3 in stromule-proximal complexes in Arabidopsis. These associations suggest a mechanistic link between stromule biogenesis and WHY1-associated plastid RNA modification. This process is increasingly recognized as being essential for retrograde signal fidelity, chloroplast biogenesis, and nutrient-responsive root development across various species [33,38,41,43,91,93,94,95]. Therefore, we speculate that WHY1 and its protein complex play a role in stress resilience by forming various stromules in response to environmental stress. Further research is needed to provide genetic and biochemical evidence.

Taken together, these findings establish a spatially resolved interaction map of the WHY1-centered protein network that connects the chloroplasts and the nuclear envelope in Arabidopsis. Under normal growth conditions involving light, WHY1 promotes the localized enrichment of molecular machinery involved in mRNA binding, plastid RNA processing, and translational regulation, as well as the enrichment of tubular stromules. In contrast, under stress from the photosynthetic inhibitor DBMIB, WHY1 reorganizes its interactions towards the formation of vesicular stromule-like structures, facilitating the recruitment of factors that modulate ribosomal stability and protein homeostasis. These context-dependent stromule morphology-coupled assemblies position WHY1 as a central integrator that coordinates the developmental fate of the chloroplast and retrograde signaling output.

## 4. Materials and Methods

### 4.1. Materials

The wild-type (WT) *Arabidopsis* Col-0 ecotype and a series of transgenic *Arabidopsis* seeds were stratified at 4 °C in the dark for 2 days, then grown at 22 °C under a 16 h/8 h light/dark cycle, a light intensity of 80 μmol/m^2^/s, and 65% humidity in a climate chamber.

The mutant lines include: (1) *why1why3* (originally kindly provided by the Brission group. (2) *oeWHY1–GFP*, *P_WHIRLY1_-nWHY1–HA/GFP*, and *P_WHIRLY1_-WHY1–HA/GFP* lines were previously generated [23,39,61], and the oeGFP (35S:GFP) and oeCTP-GFP (35S promoter driving a chloroplast transit peptide derived from the *Rubisco small subunit* gene fused to GFP) lines were screened for GFP fluorescence signals and immunoblotted with an anti-GFP antibody to determine moderate levels of protein expression (Figure S3).

Treatment experiments: A single representative line was chosen to conduct GFP–GBP affinity proteomics. Six-week-old T2 generation transgenic plants, including *oeWHY1-GFP*, *oeGFP*, and *oeCTP-GFP* plants grown after the dark cycle, were set up under three treatments: (1) extended darkness treatment for 9 h; (2) returned to 9 h of normal growth light conditions at room temperature; and (3) after 7 h of light growth, treated with 12 μM DBMIB for 2 h. Three and four biological replicates, with 0.4 g of the fifth–eighth leaves of each plant, were sampled. The materials were then frozen and ground into a fine powder using liquid nitrogen for protein isolation. At the same time, the fresh leaves were collected for observation under a confocal microscope and for sample preparation using a transmission electron microscope.

### 4.2. Plasmid Construction and Plant Transformation

All constructs were generated according to the manual of the In-Fusion cloning kit (Clon-Express II One Step Cloning kit, Vazyme, Nanjing, China, C112-01). To generate constructs for BiFC assays, full-length coding sequences of WHY1 and candidate genes (*MORF2*, *CMS1*, *TUB3*, *TUBB9*, *NRIP1*, *TRXz*, *GRF6,* and *NOT3)* without the stop codon were inserted into pRTVcVC (C-terminal fused with C-truncated Venus fluorescent protein) and pRTVcVN (C-terminal fused with N-truncated Venus fluorescent protein), respectively. The primers used to generate BiFC constructs are listed in Table S4. To generate constructs for yeast two-hybrid assays, full-length coding sequences of WHY1 and candidate genes (*MORF2*, *CMS1*, *TUB3*, *TUBB9*, *NRIP1*, *TRXz*, *GRF6,* and *NOT3)* without the stop codon were inserted into the pGAD427 vector and the pGDBT7 vector, respectively, using In-Fusion cloning (Clon-Express II One Step Cloning kit, Vazyme, C112-01). The primers are listed in Table S4. To prepare CoIP vectors, the full-length coding region of *NRIP1* with a C-terminal HA tag was amplified using gene-specific primers (Table S4) and cloned into pDONR201. This construct was subsequently recombined into the expression vector p2GW7 (Life Technologies, Darmstadt, Germany) using Gateway^®^ cloning technology. The plasmid p2HAGW7 (N-terminal HA-tag) served as an empty vector control.

### 4.3. WHY1–GFP–GBP Affinity-Based Pulldown Proteomics Analysis

The WHY1–GFP-based affinity purification of GBP proteins has been previously described [66]. First, 0.2 g of leaves were collected and ground into a fine powder in liquid nitrogen. The total protein was extracted with 1 mL of a protein extraction buffer (50 mM Tris (pH 7.5), 150 mM NaCl, 0.5% Triton X-100, 0.5% Nonidet P-40, 0.5% sodium deoxycholate, protease inhibitor cocktail, and 40 μm MG132). After a brief period of centrifugation to pellet debris, 10 µL of GFP-trap beads (ChromoTek, gtak, Planegg-Martinsried, Germany) were added to the total protein extract, which was then incubated at 4 °C for 10 h with gentle shaking. The beads were then washed five times with protein extraction buffer; GFP Agarose gel beads were finally extracted with the SDT elution buffer to obtain IP proteins. The amount of protein was quantified with the BCA Protein Assay Kit (Bio-Rad, Hercules, CA, USA). All the protein samples were packed separately and stored at −80 °C for proteomic mass spectrometry analysis. IP proteins were first immunodetected with an antibody against GFP (TransGen, Beijing, China, HT801, 1:1000 dilution).

Then, 20 µg of IP proteins for each sample were mixed with 5X loading buffer and boiled for 5 min. The proteins were separated on a 12.5% SDS–PAGE gel (constant current 14 mA, 90 min). Protein bands were visualized by Coomassie Blue R-250 staining. Protein digestion was performed with trypsin. The digest peptides of each sample were desalted on C18 Cartridges (Empore™ SPE Cartridges C18 (standard density), Oxford, PA, USA, bed I.D. 7 mm, volume 3 mL, Sigma, St. Louis, MO, USA), concentrated using vacuum centrifugation, and reconstituted in 40 µL of 0.1% (*v*/*v*) formic acid.

High-pH reversed-phase method: The Pierce high-pH reversed-phase fractionation kit (Thermo scientific, Waltham, MA, USA) was used to fractionate the peptides of each sample into 6 fractions (or 10 fractions as determined by the project proposal) using an increasing acetonitrile step-gradient elution according to the instructions. The resulting peptides were collected using an aqueous solution of 0.1% formic acid in 84% acetonitrile, and lyophilized using a Speedvac before LC–MS/MS analysis. MS/MS spectra were searched in “uniprot”, a public database (http://www.uniprot.org/); “Arabidopsis”, organism species; “76,417”, the number of sequences; peptide FDR ≤ 0.01; and against the TAIR10 database using Proteome Discoverer (v.1.4). To specifically select enriched protein candidates, ratiometric analysis was conducted using the peptide peak areas (label-free quantification, LFQ values) from the experimental and control samples and the DEP package (v.1.24.0) in R (v.4.3.2). The candidates were further filtered on the basis of the normalized peptide spectrum match (PSM) value. On the basis of WHY1, known interactors of each bait protein, and other data quality parameters, the cut-off values for LFQ and PSM data were specified in each case.

### 4.4. Bioinformatic Analysis

#### 4.4.1. Cluster Analysis of Modified Peptides

Cluster 3.0 (http://bonsai.hgc.jp/~mdehoon/software/cluster/software.htm, accessed on 30 August 2019); C Clustering Library version 1.59) and Java Treeview 3.0 software (http://jtreeview.sourceforge.net, accessed on 30 August 2019) were used to perform hierarchical clustering analysis. The Euclidean distance algorithm for similarity measurements and the average linkage clustering algorithm (clustering uses the centroids of the observations) for clustering were selected when performing hierarchical clustering. A heat map is presented as a visual aid in addition to the dendrogram.

#### 4.4.2. Motif Analysis and Subcellular Localization

The motifs were analyzed using MEME (http://meme-suite.org, version 5.5.8, accessed on 30 August 2019). We extracted the amino acid sequences containing the modified site and six upstream/downstream amino acids from the modified site (13 amino acid sites in total). These sequences were used to predict motifs in this study (parameters: width: 13, occurrences:20, background: species). CELLO (http://cello.life.nctu.edu.tw/, version 2004, accessed on 30 August 2019), which is a multi-class SVM classification system, was used to predict protein subcellular localization.

#### 4.4.3. GO Annotation

The protein sequences of the selected differentially expressed proteins were locally searched using the NCBI BLAST+ client software (ncbi-blast-2.2.28+-win32.exe) and InterProScan to find homologue sequences. Then, Gene Ontology (GO) terms were mapped, and sequences were annotated using the software program Blast2GO version 6.0. Sample variation was estimated in the dataset using the rlog (DESeqDataSet, blind = TRUE) transform function included in the package, followed by the plotPCA() function, and visualized using the R package ggplot2 version 3.5.1.

#### 4.4.4. Enrichment Analysis

Enrichment analyses were applied based on Fisher’s exact test, considering whole quantified proteins as the background dataset. Benjamini–Hochberg correction for multiple testing was further applied to adjust derived *p*-values. Gene ontology (GO) and KEGG enrichment analyses were performed using the graphical enrichment tool Shiny Go v0.80. Gene function categories were selected with an enrichment FDR (false discovery rate) of *p* < 0.05.

#### 4.4.5. Protein–Protein Interaction Analysis and Prediction of IDRs

Protein–protein interactions with experimental support were retrieved from the STRING database (https://string-db.org/, version 12.0, accessed on 30 August 2019) and visualized using Cytoscape (v.3.9.1). The prediction of IDRs in WHY1-associated proteins was performed using the Database of Disordered Protein Predictions (D2P2) (https://d2p2.pro/ accessed on 25 July 2024) [96] and the VSL2 predictor from PONDR (http://www.pondr.com/ accessed on 20 August 2023) [97]. The RNA-binding proteome in *Arabidopsis* was retrieved from a previous publication [98]. The prediction of transmembrane domains in proteins within the Arabidopsis genome (https://www.arabidopsis.org/, accessed on 20 July 2024) was conducted using TMHMM-2.0 (https://services.healthtech.dtu.dk/services/TMHMM-2.0/ accessed on 20 July 2024).

### 4.5. Bimolecular Fluorescence Complementation Assay

The plasmids pRTVcVN and pRTVcVC were used for the Venus–BiFC assay [99]. For transient expression, different combinations of BiFC constructs were co-transformed into *Arabidopsis* protoplasts obtained from 4-week-old seedlings, as described previously [100]. After incubation for 16h, the transformed protoplasts were examined using a Leica TCS SP8 confocal laser scanning microscope [100]. The excitation and detection wavelengths for Venus were 514 nm for excitation and 520–570 nm for detection.

### 4.6. Co-Immunoprecipitation Assay

Constructs expressing the corresponding NRIP1 and VIPP1 HA-tagged proteins were transformed into Arabidopsis protoplasts prepared from the leaves of four-week-old oeWHY1-GFP plants. After 16 h of incubation, the transfected protoplasts were harvested for Co-IP and immunoblotting assays, following the protocol described previously [100]. The HA-tagged proteins were immunoprecipitated using anti-HA antibody-coupled beads (anti-HA affinity matrix, Roche, Basel, Switzerland), while the GFP-tagged proteins were immunoprecipitated using anti-GFP affinity beads (SA070001; Smart-Lifesciences, Changzhou, China). The immunoprecipitated proteins were then separated via 12% SDS–PAGE, blotted, and analyzed by immunoblotting using anti-GFP (TransGen, HT801, 1:1000 dilution) and Anti-HA-Peroxidase (Roche, 3F10, 1:500 dilution) antibodies.

### 4.7. Fluorescence Imaging and FRAP Analysis

As previously described. Briefly, FRAP data of expressed condensates in *Arabidopsis* leaf epidermal cells of *oeWHY1-GFP* plants under dark, light, and DBMIB treatments were obtained on a Leica TCS SP8 confocal microscope using the ×63 objective. Condensates were bleached using a laser intensity of 100% at 485 nm and 562 nm with 100 iterations. After photobleaching, images were continuously captured for 10 min at 5 s intervals using NIS-Elements software. Mean fluorescence intensities of the bleached region were normalized; analysis was carried out using ImageJ, and the recovery curve was generated using GraphPad Prism 10.

### 4.8. Fluorescence Microscopy Observation

The fifth–eighth leaves of six-week-old dark-, light-, and DBMIB-treated *oeWHY1–GFP Arabidopsis* plants were used to observe the stromule structures in epidermal cells and mesophyll cells under a Leica TCS SP8 confocal microscope using the ×63 objective, according to the method described by Kwok and Hanson (2004) [101].

### 4.9. Electron Microscopy Observation

The fifth leaves of six-week-old Arabidopsis plants were sectioned into small pieces, transferred into a cell-stabilizing solution containing 25 mM HEPESKOH (pH 6.5), 5 mM KCl, 1 mM CaCl_2_, and 1 M mannitol or 0.3 M mannitol, respectively. Samples were fixed and embedded in LR white resin (Polysciences Inc., Warrington, PA, USA). Sections were stained with uranyl acetate and lead citrate. A Hitachi TEM system HC-1 HT7800 transmission electron microscope (Center of analysis and detection of Fujian Agriculture and Forestry University) was used. Images were taken at an accelerating voltage of 80 kV. All diameter measurements were made using ImageJ (https://imagej.net/ij/, version 1.54t, accessed on 2 November 2023) on at least ten sample micrographs from three independent experiments.

### 4.10. Statistical Analysis

Data in all figures were determined by at least three biological replicates. Student’s *t*-test and multiple-factor one-way ANOVA tests were used to determine statistical significance.

## Figures and Tables

**Figure 1:**
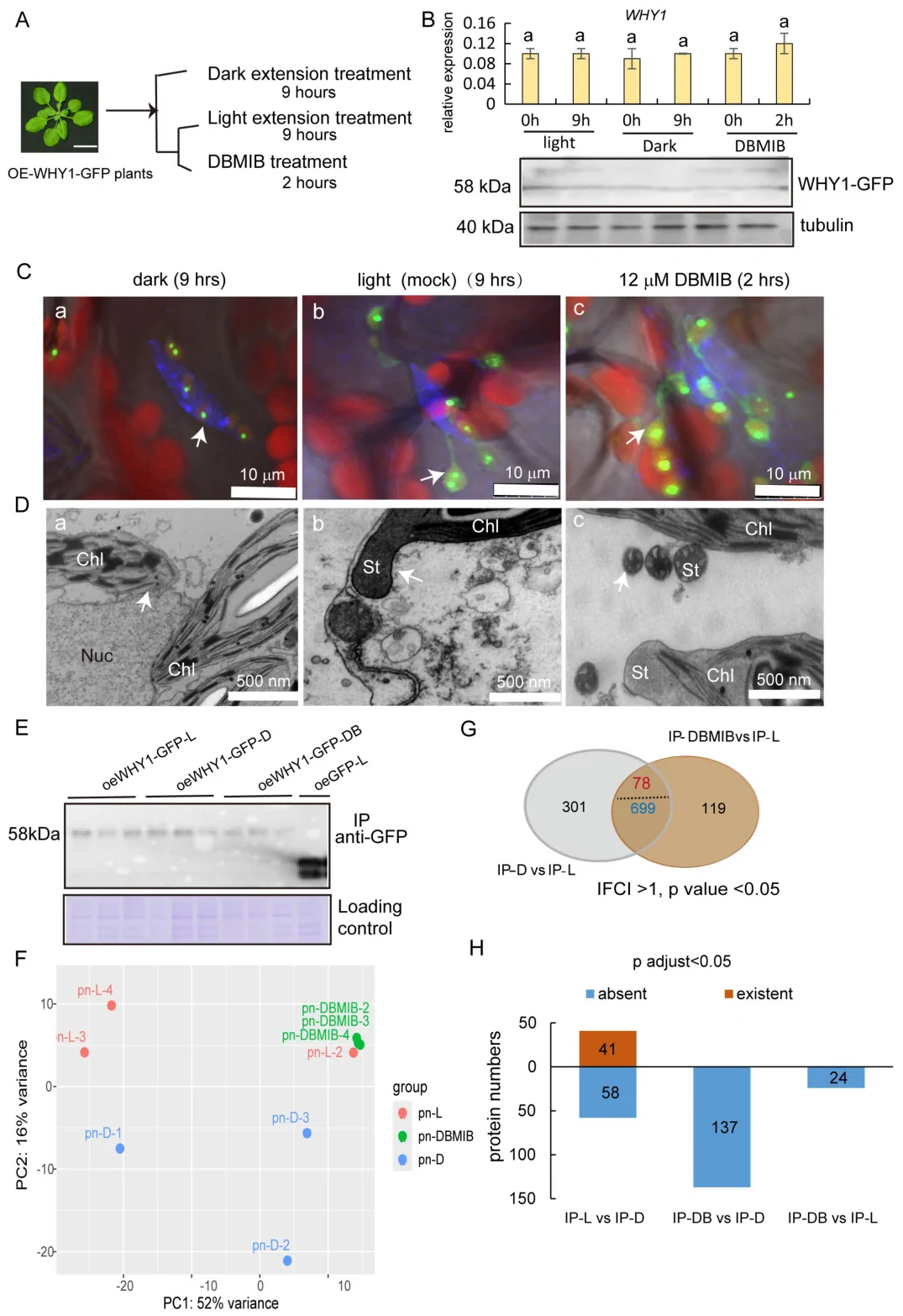
Proteomic analysis of WHY1-associated stromule formation under light and DBMIB treatment conditions. (**A**) Sample treatment design; (**B**) transcript level of WHY1 in the *oeWHY1–GFP* plants under light, dark, and 12 μM DBMIB treatments (top). Different letters indicate significant differences (*n* = 3 × 3); the protein level of *oeWHY1–GFP* was analyzed using an antibody against GFP in Western blotting (bottom). (**C**) Confocal observation of stromule formation under dark (a), light (b), and DBMIB (c) treatment conditions. Scale bar = 10 μm; arrowheads indicate the WHY1–GFP complex; blue indicates DAPI staining, and red indicates chlorophyll in chloroplasts. (**D**) Stromule-like structure formation observed under transmission electron microscopy under dark (a), light (b), and DBMIB (c) treatment conditions. Scale bar = 500 nm. The arrowheads indicate different types of chloroplast–nucleus connections: direct touch (a), tubular stromules (b), and vesicular-like stromules (c). (**E**) IP detection using Western blotting with an antibody against GFP to maintain the same level of WHY1 protein as in the IPs. (**F**) PCA test for nine samples (three treatments, three replicates). (**G**) Venn diagram showing the overlap between the WHY1 pull-downed proteins in IP-DBMIB vs. IP-L and IP-D vs. IP-L (|Log2 FC| >1, *p* < 0.05). (**H**) DEP assembly (*p* adj < 0.05) in IP-L relative to IP-D, IP-DBMIB relative to IP-D, and IP-DBMIB relative to IP-L.

**Figure 2:**
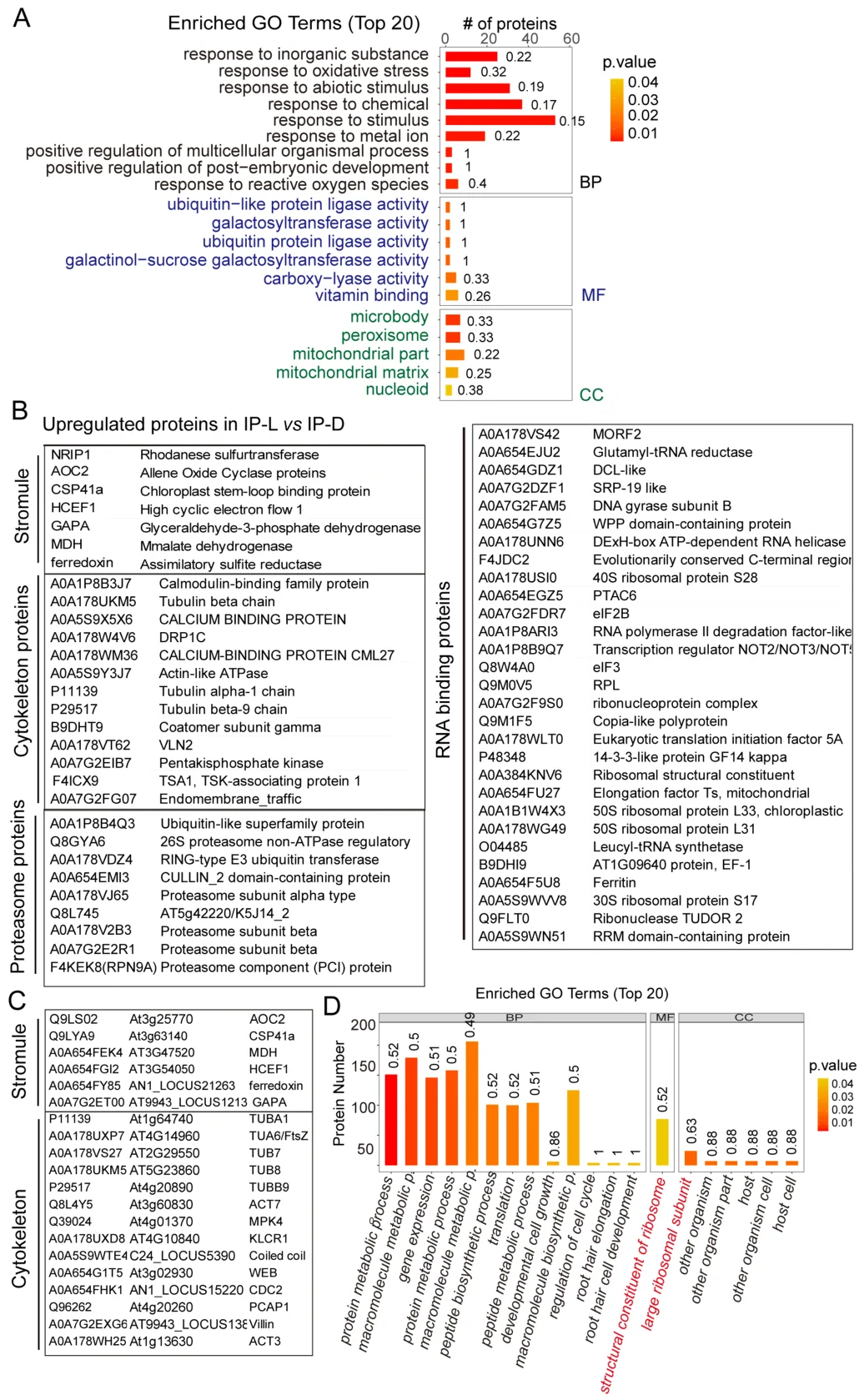
GO analysis of the WHY1 pulldown protein assembly of IP-L vs. IP-D and IP-DBMIB vs. IP-L (|log2FC| ≥1, *p*-value < 0.05). (**A**) The top 20 enriched GO terms of the DEP assembly of IP-L vs. IP-L. The percentage of enriched proteins relative to the total number of proteins in the GO term is shown at the top of the bars. (**B**) A list of the DEPs enriched in GO terms, including stromule proteins, RNA-binding proteins, cytoskeletal proteins, proteasome proteins, and others. (**C**) Several stromule proteins and cytoskeletal proteins enriched in the DEP assembly of IP-DBMIB vs. IP-D (|log2FC| ≥ 1, *p*-value < 0.05). (**D**) GO analysis of the DEP assembly of IP-DBMIB vs. IP-D reveals enrichment of the structural component of the ribosome. The percentage of enriched proteins relative to the total number of enriched proteins in the GO term is shown at the top of the bars.

**Figure 3:**
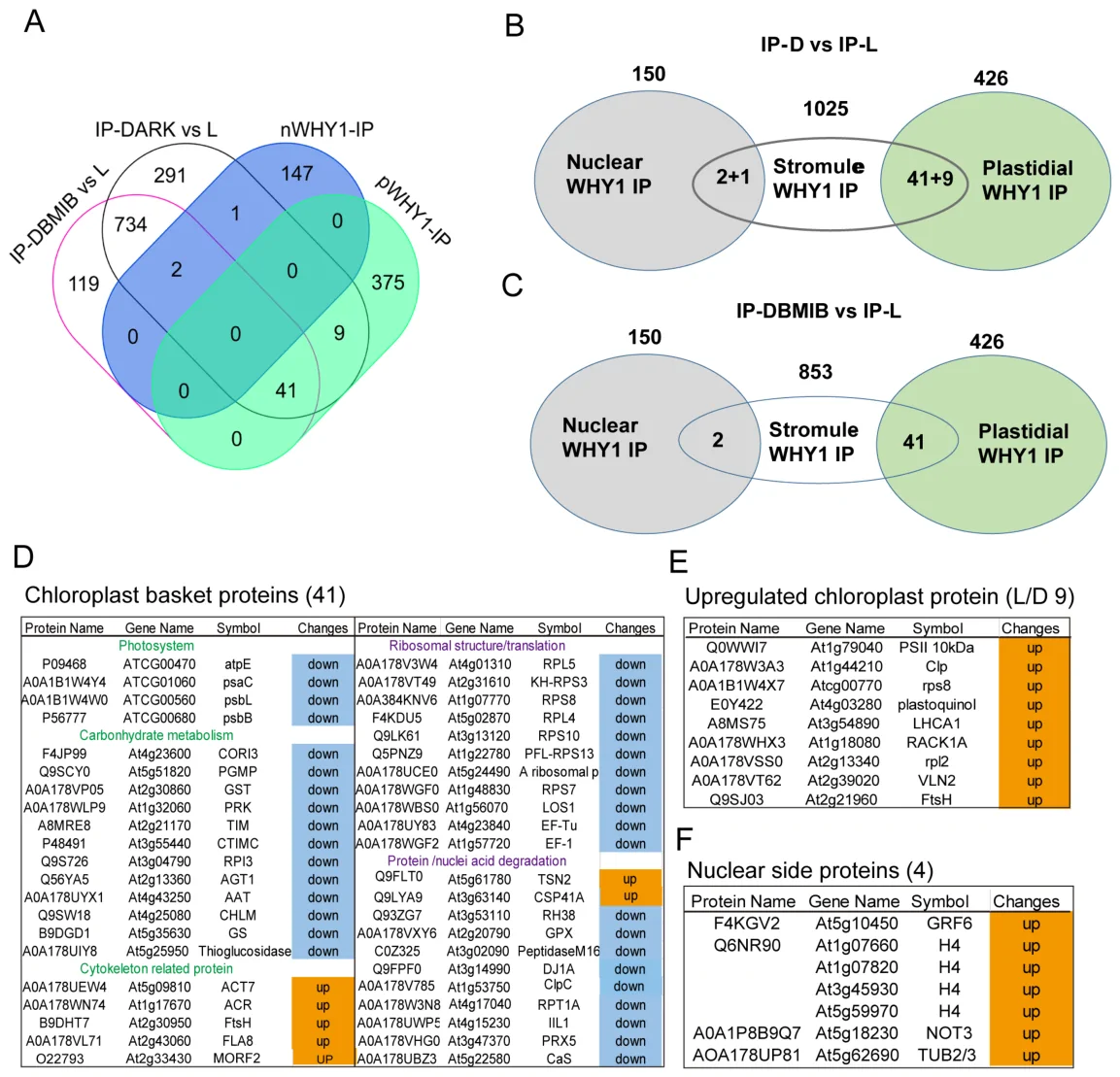
A landscape of the WHY1 pulldown proteome between the chloroplast envelope and the nuclear envelope. (**A**). Venn analysis of the WHY1 interactome protein assembly (IP-D/IP-L and IP-DBMIB/IP-L) with the local IP-pWHY1 and IP-nWHY1 proteomes (Table S4) (**B**). The distribution of the WHY1 pulldown proteome assembly in the chloroplast side and nuclear sides from IP-D/IP-L (**C**). The distribution of the WHY1 pulldown proteome assembly in the chloroplast and nuclear sides from IP-DBMIB/IP-L; D–F. The lists of proteins in the chloroplast from IP-D/IP-L and IP-DBMIB/IP-L (**D**). Additional proteins on the chloroplast side from IP-D/IP-L (**E**), and proteins in the nucleus (**F**). blue indicates down regulated, oragen indicates upregulated.

**Figure 4:**
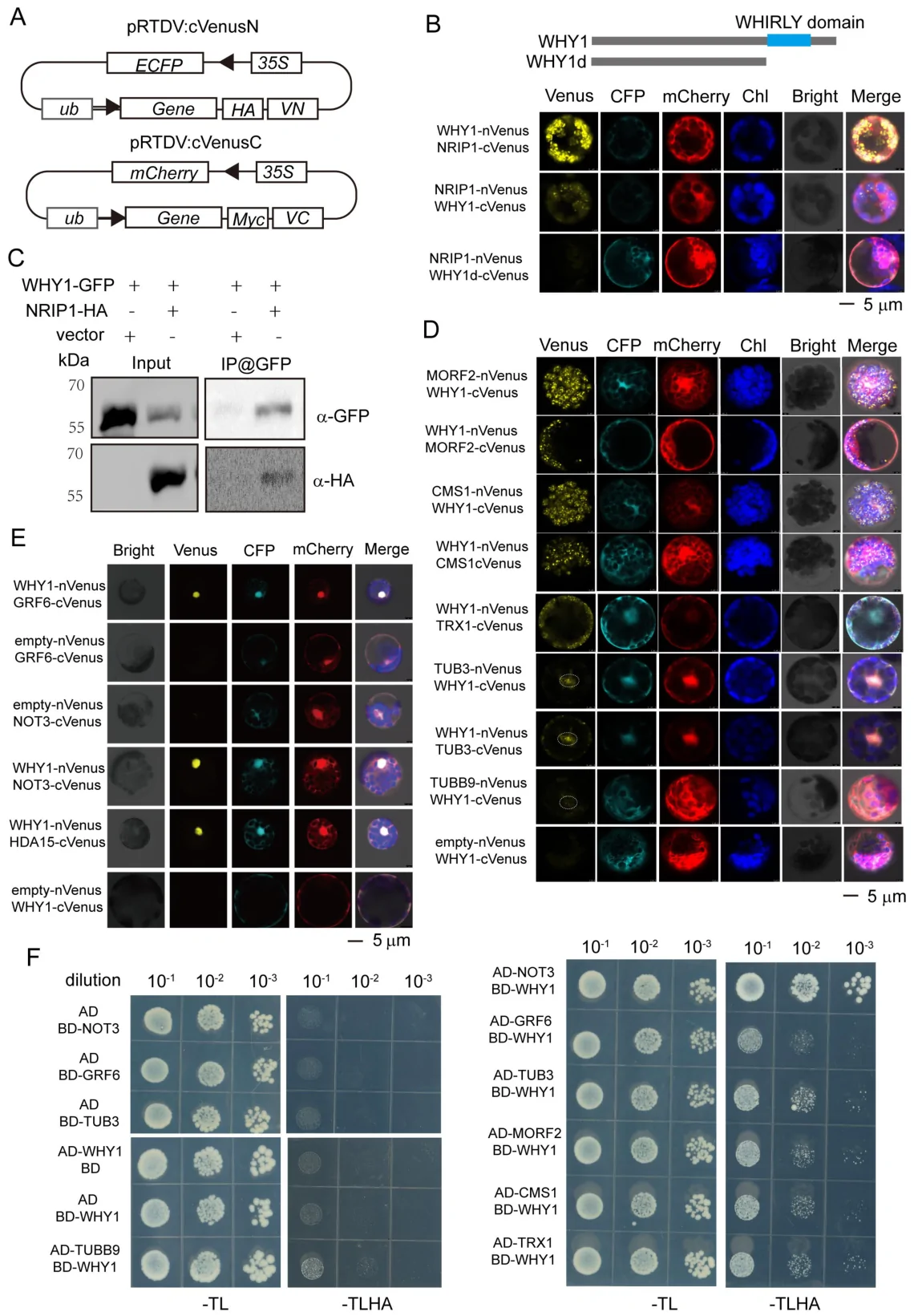
Confirmation of WHY1-interacting proteins using bimolecular fluorescence complementation (BiFC) and yeast two-hybrid assays. (**A**). Schematic of the plasmid used in the BiFC assay (**B**). The WHY1 protein interacts with the NRIP1 protein in chloroplasts in the Venus–BiFC assay. WHY1: full-length WHY1; WHY1d: deleted WHIRLY domain. (**C**). The WHY1 protein interacts with NRIP1 in the Co-IP assay. The antibody against GFP was used for immunoprecipitation, and the antibody against HA was used to detect the IP protein. An empty vector was used as a negative control. (**D**). The WHY1 protein interacts with MORF2, TRX1, and CMS1 in the chloroplast basket, and with TUB3 in the nucleus, but not with TUBB9. (**E**). WHY1 interacts with GRF6, NOT3, and HDA15 (positive control) in the nucleus. Scale bars = 5 μm. (**F**). The yeast two-hybrid assay confirms the interactions.

**Figure 5:**
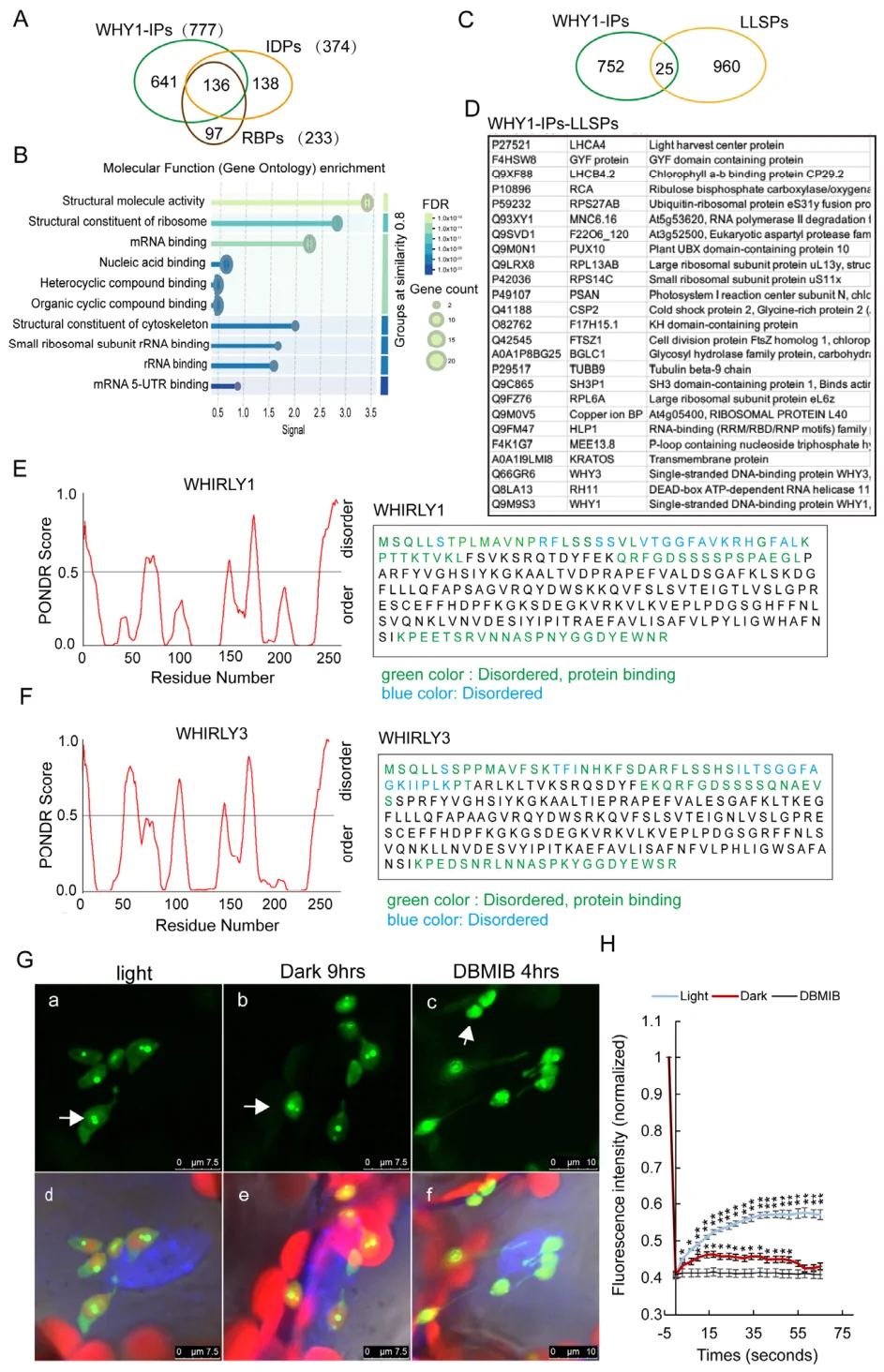
Phase separation phenomena exist in the WHY1-associated protein assembly. (**A**). Venn analysis of WHY1-associated proteins that enriched in non-membrane-bound organelles component with IDP protein assemblies in the genome. (**B**). GO analysis of 136 IDP-RBPs reveals that non-membrane-bound organelle proteins are enriched in RNA metabolism. (**C**). Venn analysis of WHY1-associated proteins with LLSP protein assemblies in plants [62] (**D**). List of WHY1-associated LLSP1. (**E**,**F**). WHY1 and WHY3 proteins are predicted to be IDR-containing (IDPs) by the PONDR program (http://pondr.com/ accessed on 20 August 2023) and protein se-quence prediction (**G**). Fluorescence microscopy observation showing the WHY1 complex in light and DBMIB-treated conditions. The arrowhead indicates the WHY1–GFP protein complex forming an aggregated structure (a,b) or a diffused structure (c); (d,e,f) are the merged photos of (a,b,c), respectively, fluorescent signal (green) with chloroplast (red) with DAPI staining (blue). (**H**). Flu-orescence recovery of the aggregated structure in light and dark conditions, but not in DBMIB-treated samples after photobleaching (FRAP) analysis (*n* = 6). A Student’s *t*-test was used to determine significance relative to DBMIB treatment. * *p* < 0.05, ** *p* < 0.01, *n* = 6.

**Figure 6:**
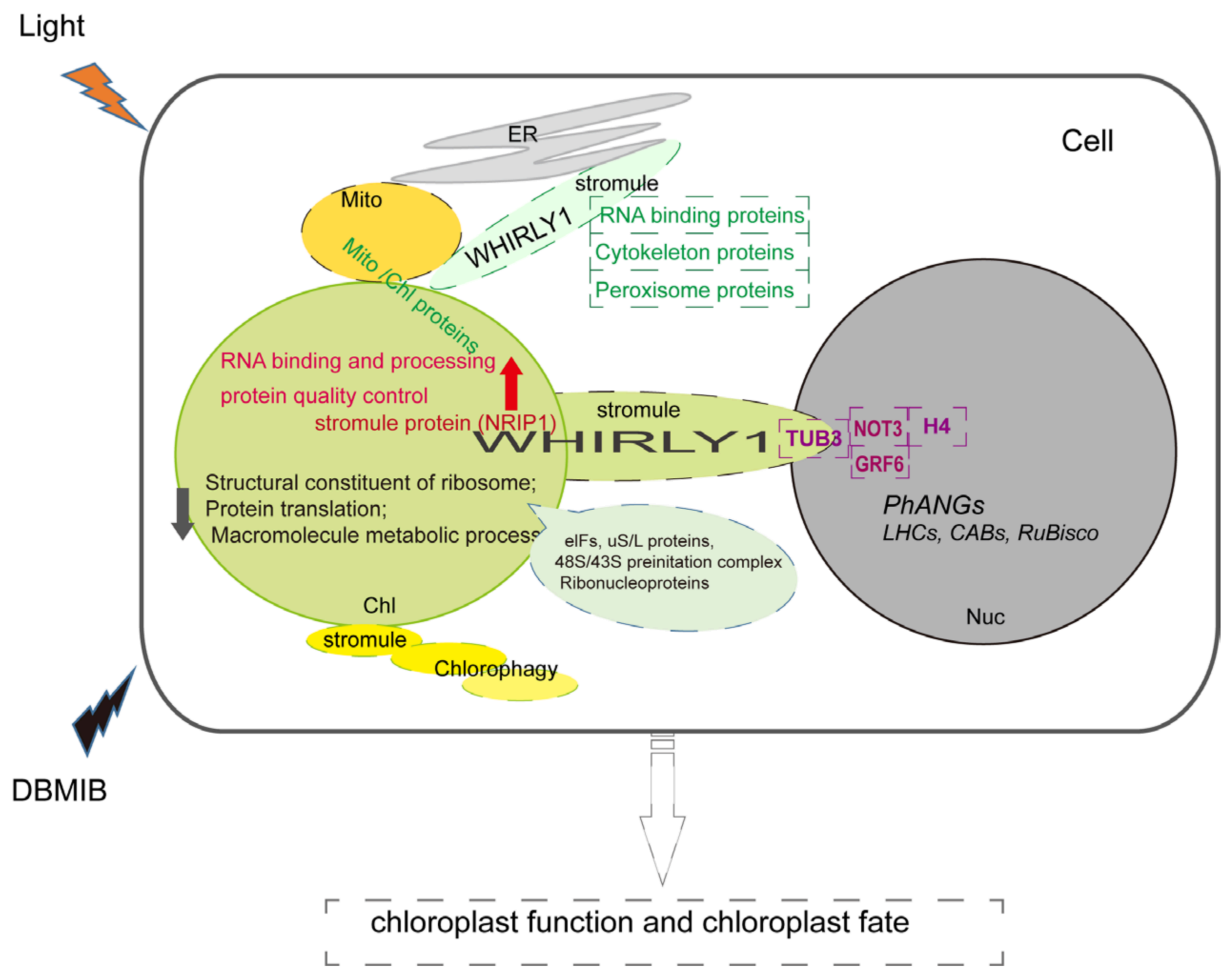
A spatially resolved interaction map of the WHY1-centered protein network that connects chloroplasts and the nuclear envelope through stromule formation in *Arabidopsis*. Under normal light conditions, the WHY1-associated protein complex enriches the molecular machinery involved in mRNA binding, plastid RNA processing, and translational regulation. These processes are associated with the formation of tubular stromules. In contrast, under DBMIB stress, the WHY1-associated protein complex undergoes reorganization, which is connected with the formation of vesicular, stromule-like structures. This facilitates the recruitment of factors that modulate ribosomal stability and protein homeostasis. These context-dependent, morphology-coupled assemblies of stromule-related complexes position WHY1 as a central integrator involved in the coordination of the developmental fate of the chloroplast and retrograde signaling output. The red arrowhead indicates the molecular function terms related to accumulated proteins, and the gray arrowhead indicates the molecular function terms related to dismissed proteins. The broken lines indicate hypothetical models.

**Table 1:** The list of WHY1-associated stromule-related proteins in this dataset.

Protein ID	Gene Name	GO Term 0010319	Annotation	Refs.
A0A654FGI2	*HCEF1*	stromule	High cyclic electron flow 1	[46]
A0A384L5D6	*VIPP1*	stromule	Protein thylakoid formation 1, Vesicle-induced protein 1	[47,48]
A0A5S9YEA7	*PSB29/* *THF1*	stromule	Photosystem II reaction center, Thiamine thiazole synthase	[47,48,49]
A0A654FLZ8	*PAP-FIB*	stromule	Fibrillin	[46]
Q9LS02	*AOC2*	stromule	Allene oxide cyclase proteins	[46,50,51]
Q9LYA9	*CSP41A*	stromule	Chloroplast stem-loop binding protein of 41 kDa A	[45]
A0A654FY85	*Ferredoxin*	stromule	Assimilatory sulfite reductase	[52]
A0A5S9WM95 A0A7G2ET00	*GAPA3*	stromule	Glyceraldehyde-3-phosphate dehydrogenase	[53,54]
A0A654FEK4	*MD*	stromule	Malate dehydrogenase	[55,56]
A0A654ECJ1	*AOR*	stromule	Alkenal/One Oxidoreductase	[57,58]

**Table 2:** The list of differentially expressed proteins in *why1why3* vs. WT.

Protein Name	Gene Locus	Sub Loc Predicted by WoLF PSORT *	Regulation
TROL(thylakoid rhodanase-like protein)	*AT4g01050*	Chloroplast	up
Malate dehydrogenase	*AT3g47520*	Chloroplast	up
Thioredoxin-like protein CDSP32	*AT1g76080*	Chloroplast	up
ATP-dependent Clp protease proteolytic subunit-related protein 3	*AT1g09130*	Chloroplast	up
Thylakoid lumenal 29 kDa protein, APX4	*AT4g09010*	plastid	up
Peptide methionine sulfoxide reductase A4	*AT4g25130*	plastid	up
Beta-amylase 3	*AT4g17090*	Chloroplast	up
L-galactono-1,4-lactone dehydrogenase	*AT3g47930*	mitochondrion	up
Formate dehydrogenase	*AT5g14780*	Chloroplast	up
Glutamate-glyoxylate aminotransferase 1	*AT1g23310*	peroxisme	up
12-oxophytodienoate reductase 3	*AT2g06050*	peroxisme	up
Proteasome subunit alpha type-6-B	*AT2g05840*	cyt/nuc	up
NADP-dependent malic enzyme 2	*AT5g11670*	cyt	up
Asparagine synthetase 2	*AT5g65010*	cyt	up
Pyridoxal 5′-phosphate synthase subunit	*AT2g38230*	cyt	up
AT4G23670 protein	*AT4g23670*	vacuole	up
PSRP3	*ATCg00800*	plastid	down
PSRP5	*AT2g33800*	plastid	down
VIPP1	*AT1g65260*	plastid	down
Acetylglutamate kinase	*AT3g57560*	plastid	down
Beta carbonic anhydrase 1	*AT3g01500*	plastid	down
Ribosome-recycling factor	*AT3g63190*	plastid	down
Ribulose bisphosphate carboxylase small chain 1B	*AT5g38430*	chloroplast	down
Ribulose bisphosphate carboxylase small chain 1A	*AT1g67090*	chloroplast	down
Ribulose bisphosphate carboxylase large chain	*ATCG00490*	chloroplast	down
Chlorophyll a-b binding protein	*AT3g54890.1*	plastid	down
F16A14.27	*AT1g13930*	chloroplast	down
GRP7	*AT2g21660*	cyt/nuc	down
Nascent polypeptide-associated complex subunit beta	*AT1g18780*	cyt/nuc	down
Glutathione S-transferase F7	*AT1g02920*	cyt	down
Kinesin-like calmodulin-binding protein	*AT5g65930*	cyt	down
Peptidyl-prolyl cis-trans isomerase CYP19-1	*AT2g16600*	cyt	down
Remorin	*AT2g45820*	plasmic membrane	down
V-type proton ATPase subunit G1	*AT3g01390*	vesicle	down
GDSL esterase/lipase At1g29670	*AT1g29670*	cyt	down

* Website: https://www.genscript.com/wolf-psort.html (accessed on 26 July 2024).

## Data Availability

All data are available in the main text or the Supplementary Materials.

## Supplementary Materials

The following supporting information can be downloaded at: https://www.mdpi.com/article/10.3390/plants15192930/s1. Supplementary Figures: Figure S1: The expression level of WHY1 under light, dark, and DBMIB treatment conditions. Figure S2: The procedure of WHY1–GFP immunoprecipitation–mass spectrum analysis. Figure S3: Detection of IP-nWHY1 and IP-pWHY1 relative to IP-GFP and IP-CTP-GFP. Figure S4: The protein-associated network of WHY1-IPs-IDPs by string analysis and their functional enrichment. Supplementary Tables: Table S1: The list of known stromule-related proteins. Table S2: The IP-WHY1 DEPs (*p* < 0.05) in IP-D/IP-L. Table S3: The IP-WHY1 DEPs (*p* < 0.05) in IP-DBMIB/IP-L or IP-D. Table S4: The list of primers used in this study. Supplementary Datasets: Dataset S1: All datasets of IP-D/IP-L, IP-DBMIB/IP-L, and IP-DBMIB/IP-D in oeWHY1-GFP relative to IP-GFP. Dataset S2: IP-D/IP-L-GO. Dataset S3: IP-DBMIB/IP-L-GO. Dataset S4: IP-pWHY1 and IP-nWHY1. Dataset S5: LLSP-associated proteins.
