# Supplementary material for: WHIRLY1 Interactome Profiling Reveals the Chloroplast Fate Correlation with Stromule-like Morphologies in Arabidopsis Leaves Under Dark and DBMIB Stress Conditions
Source: Plants (Basel). 2026 Sep 24;15(19):2930. doi: 10.3390/plants15192930 (PMC13666948; doi:10.3390/plants15192930)
Supplement: Supplementary file 1 [file plants-15-02930-s001.zip › Supplementary FigS1-4-TableS1-4-Plants.pdf]

Supplementary information

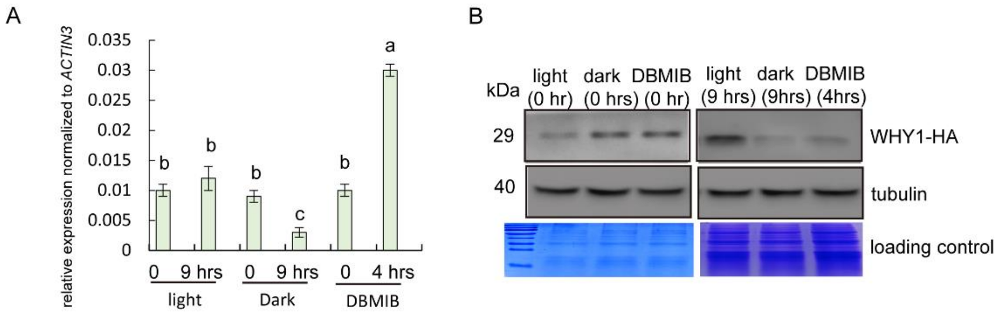

Figure S1 The expression level of WHY1 in the P_{WHY1}-*WHY1-HA* plants under the treatment of light, dark, and 12 μM DBMIB. A. transcript level, the different letter indicates significant different (n=3x3); B the protein level, antibody against HA was used to do western blotting.

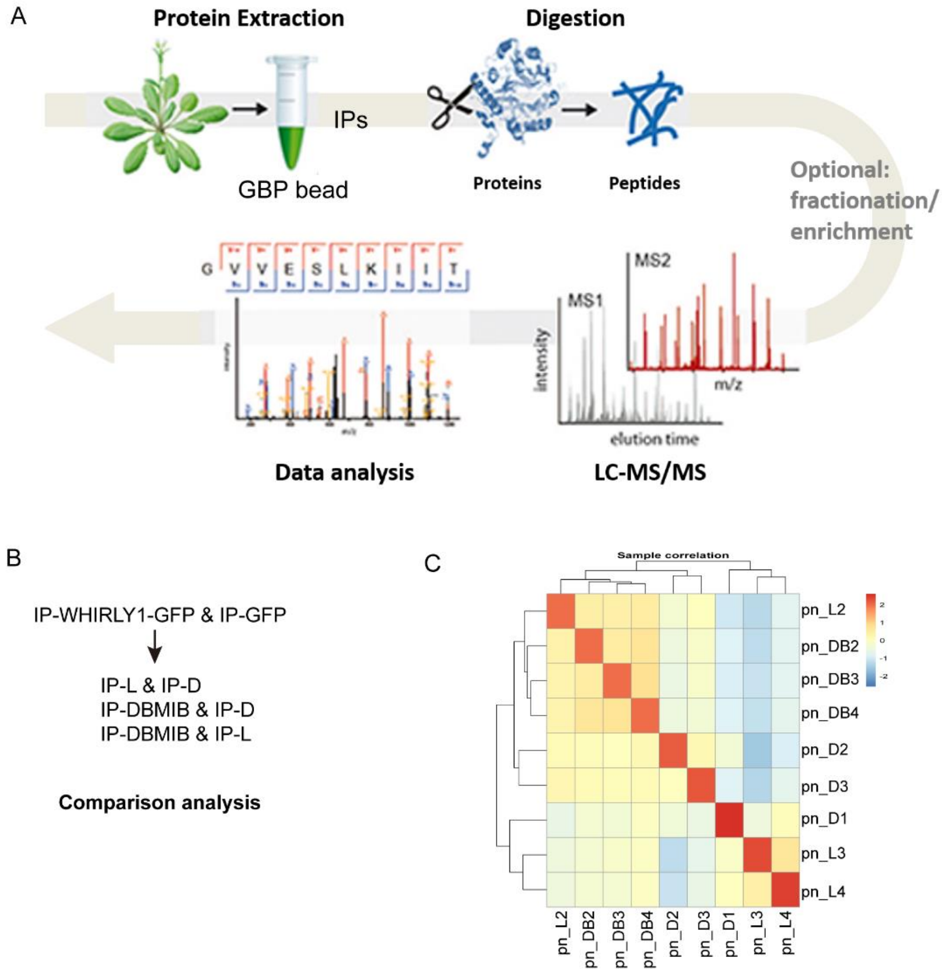

Figure S2 WHIRLY1-GFP immunoprecipitation-mass spectrum analysis.

A. the sample treatments; B.The procedure of WHIRLY1-GFP immunoprecipitation-mass spectrum analysis; B. comparison analysis; C. E Person analysis of three replicate samples.

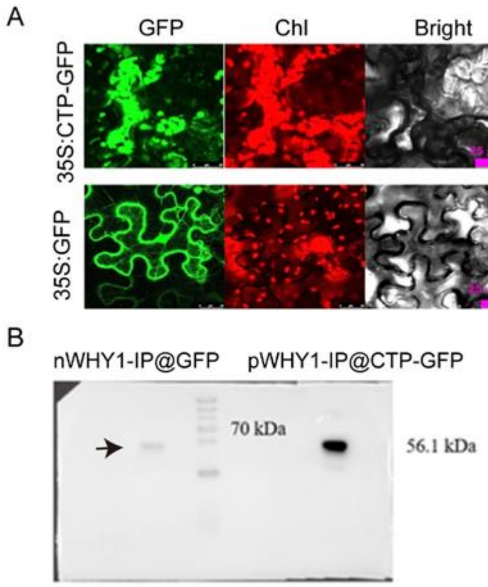

Figure S3 Identification of 35S:CTP-GFP transgenic plants.
A.GFP fluorescence detection; B. western blot detection for proteins used in AP-pulldown assay of nWHY1 and pWHY1 proteins.

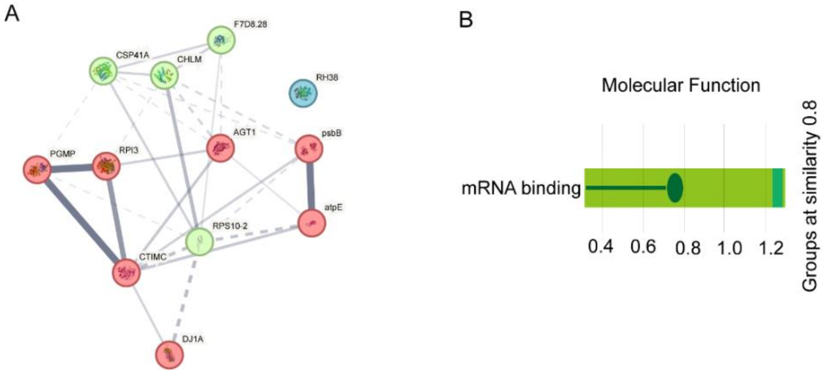

Figure S4 The LLSP proteins were enriched in theWHIRLY1 associated protein assembly.
A.String analysis ((https://string-db.org/) and visualized using Cytoscape (v.3.9.1); B. GO term of molecular function.

Supplementary Table S1 The list of known stromule proteins

Protein ID	Gene name	GO term 0010319	Annotation	Refs
**A0A654FGI2**	HCEF1	stromule	high cyclic electron flow 1	Goulas et al., 2006 (28)
**P10896**	RCA	stromule	rubisco activase	Goulas et al., 2006
**Q9SCY0**	PGM	stromule	phosphoglucomutase	Goulas et al., 2006
**A0A384L5D6**	VIPP1	stromule	Protein THYLAKOID FORMATION 1	Huang et al., 2013 (29, 30);
**A0A5S9YEA7**	PSB29/THF1	stromule	photosystem II reaction center Thiamine thiazole synthase	(28, 29, 31)
**P21240**	PCN60B	stromule	Chaperonin 60 subunit beta 1	Goulas et al., 2006;
**A0A654FLZ8**	PAP-FIB	stromule	fibrillin	Goulas et al., 2006;
	NADBP	stromule	NAD binding protein	Goulas et al., 2006;
**Q9LS02**	AOC2	stromule	allene oxide cyclase proteins	(28, 32, 33)
**P56757**	atpA	stromule	ATP synthase subunit beta	Goulas et al., 2006
**Q9LYA9**	CSP41A	stromule	Chloroplast stem-loop binding protein of 41 kDa A	(34)
**Q9SA52**	CSP41B	stromule	Chloroplast stem-loop binding protein of 41 kDa B	
**A0A654FY85**	Ferredoxin	stromule	Assimilatory sulfite reductase	(35)
**O48737**	TRXM1	stromule	Thioredoxins M1	
**Q9SAU2**	RPE	stromule	Ribulose-5-phosphate-3-epimerase	
**A0A5S9XYQ5**	CAT	stromule	catalase	
**A0A5S9WM95 A0A7G2ET00**	GAPA3	stromule	Glyceraldehyde-3-phosphate dehydrogenase	(36,37)
**A0A654FEK4**	MD	stromule	malate dehydrogenase	(38,39)
**A0A654ECJ1**	AOR	Stromule	Alkenal/One Oxidoreductase	(40,41)
**A0A7G2EEE2**	A2AR	Stromule	adenosine A2A receptor	
**Q94BT5**	Gq	Stromule	G protein alpha subunits	
**A0A178WN74**	ACR	Stromule	ACT domain-containing protein ACR, amino acid binding protein	

Table S2 The IP-WHIRLY1 DEPs (P<0.05) in IP-D/IP-L.

Protein ID	Protein Name	IP-D/IP-L
A0A5S9XD62	Thioredoxin domain-containing protein	0.481112098236561
A0A654F8M7	Protein kinase domain-containing protein	0.477523799279299
A0A5S9Y3J7	Uncharacterized protein	0.451892598442351
A0A5S9Y7L0	MADS-box domain-containing protein	0.423662179567078
A0A178WLT0	Eukaryotic translation initiation factor 5A	0.36758079490794
P48348	14-3-3-like protein GF14 kappa	0.3629396562952
A0A384KNV6	(thale cress) hypothetical protein	0.339135345499635
P11139	Tubulin alpha-1 chain	0.316909563659405
A0A654FU27	Elongation factor Ts, mitochondrial	0.287024954845368
Q9FXM2	DC 1.2 homolog (Fragment)	0.284586605006664
A0A7G2E2R1	Proteasome subunit beta	0.252750298069797
A0A1B1W4X3	50S ribosomal protein L33, chloroplastic	0.252737662560441
A0A178VJ65	Proteasome subunit alpha type	0.220507009039696
A0A178UG43	BIP2	0.213063502384049
Q8LCW6	Similar to late embryogenesis abundant proteins	0.203046762861676
A0A178VIW5	PR 65	0.201201906748339
A0A1P8BCE3	Octicosapeptide/Phox/Bem1p family protein	0.195911977551322
A0A178WG49	50S ribosomal protein L31	0.194340050709908
Q9M0Y0	Uncharacterized protein AT4g05150	0.189147841290656
A0A178V2B3	Proteasome subunit beta	0.185509499499289
A0A178WBT3	EDA10	0.184660566077889
O04485	Leucyl-tRNA synthetase	0.163000065049112
A0A178W0C4	Serine/threonine-protein phosphatase	0.161381663435147
A0A7G2FG07	(thale cress) hypothetical protein	0.149074025405169
P29517	Tubulin beta-9 chain	0.1466375357025
B9DHT9	Coatomer subunit gamma	0.136228744720026
P55737	Heat shock protein 90-2	0.134788789729137
B9DHI9	AT1G09640 protein	0.133333668585988
A0A7G2EMC9	Acetyltransferase component of pyruvate dehydrogenase complex	0.129216171333677
A0A654F5U8	Ferritin	0.127885394715513
A0A5S9WVV8	30S ribosomal protein S17, chloroplastic	0.121299662854499
A0A178VT62	VLN2	0.120517323901795
Q9FLT0	Ribonuclease TUDOR 2	0.117279817096405
Q9SIE3	(3R)-hydroxymyristoyl-[acyl-carrier-protein] dehydratase	0.112932532183022
A0A7G2EIB7	Inositol hexakisphosphate and diphosphoinositol-pentakisphosphate kinase	0.101718494271686
A0A7G2DT14	(thale cress) hypothetical protein	0.0918714109657288
Q06738	Low-temperature-induced 78 kDa protein	0.071755737504582

		7
F4KEK8	Proteasome component (PCI) domain protein	0.0518376086143101
A0A5S9YCP5	Uncharacterized protein	0.575211895050479
B2C7Y6	NRIP1	
A0A1P8B4Q3	Ubiquitin-like superfamily protein	
A0A178VS42	MORF2	
A0A1P8BBD5	Aspartokinase	
A0A1P8B3J7	Calmodulin-binding family protein	
A0A178UKM5	Tubulin beta chain	
A0A5S9X5X6	Uncharacterized protein	
A0A654EJU2	Glutamyl-tRNA reductase	
A0A178U8T0	Hsp81.3	
A0A654ELQ9	Thioredoxin domain-containing protein	
Q8GYA6	26S proteasome non-ATPase regulatory subunit 13 homolog B	
A0A654GDZ1	Uncharacterized protein	
A0A7G2E238	(thale cress) hypothetical protein	
A0A178W4V6	(thale cress) hypothetical protein	
A0A178VDZ4	RING-type E3 ubiquitin transferase	
A0A7G2DZF1	(thale cress) hypothetical protein	
A0A7G2FAM5	DNA gyrase subunit B	
A0A654G7Z5	WPP domain-containing protein	
A0A654EMI3	CULLIN_2 domain-containing protein	
A0A178UNN6	Uncharacterized protein	
A0A5S9WN51	Uncharacterized protein	
A0A178WM36	(thale cress) hypothetical protein	
F4JDC2	Evolutionarily conserved C-terminal region 2	
A0A7G2DRL9	(thale cress) hypothetical protein	
A0A178WA97	SDC1	
A0A7G2FLZ2	(thale cress) hypothetical protein	
A0A178USI0	Uncharacterized protein	
F4JP99	Tyrosine transaminase family protein	
A0A654EGZ5	(thale cress) hypothetical protein	
A0A7G2FDR7	(thale cress) hypothetical protein	
Q8L745	AT5g42220/K5J14_2	
A0A1P8ARI3	RNA polymerase II degradation factor-like protein (DUF1296)	
A0A5S9VBT6	Uncharacterized protein	
A0A1P8B9Q7	Transcription regulator NOT2/NOT3/NOT5 family protein	
Q8W4A0	Eukaryotic translation initiation factor 3 subunit M	
Q9M0V5	At4g05400	
A0A7G2F9S0	(thale cress) hypothetical protein	
Q6XWB5	Resistance protein Ei2-2 (Fragment)	
Q9M1F5	Copia-like polyprotein	

UP		
Protein ID	Protein Name	IP-D/IP-L
A0A178V7W0	RNA helicase	25.452180335023
A0A178VT47	Phosphopyruvate hydratase	19.0855850763143
A0A7G2E1Y9	(thale cress) hypothetical protein	13.7272737070127
Q0WLB0	Xyloglucan endotransglucosylase/hydrolase	13.6233376783061
Q9FM47	RNA-binding (RRM/RBD/RNP motifs) family protein	13.2794589352761
A0A178WMD1	Uncharacterized protein	12.8432264241264
A0A654EW41	Succinate--CoA ligase [ADP-forming] subunit beta, mitochondrial	10.0556546790853
A0A178V326	(thale cress) hypothetical protein	9.92931616637602
A0A5S9X6E0	RNA helicase	9.30552278757904
A0A178VS66	GRP3	9.02206951569253
Q9SE60	Methylenetetrahydrofolate reductase 1	8.84310291169382
A8MRE8	Triosephosphate isomerase	7.81488160708403
A0A1I9LQ23	Cyclophilin-like peptidyl-prolyl cis-trans isomerase family protein	7.58653351854485
Q8RX87	Probable galactinol--sucrose galactosyltransferase 6	6.97965351126537
A0A5S9VCL0	Catalase	5.96338360794847
A0A5S9Y4Y8	Uncharacterized protein	5.89794166529688
A0A654FGI2	D-fructose-1,6-bisphosphate 1-phosphohydrolase	5.49097082607336
F4HU93	L-ascorbate peroxidase	5.22175581055275
F4JZM6	Aluminum induced protein with YGL and LRDR motifs	4.96418688839115
A0A5S9WML4	Dihydrolipoyl dehydrogenase	4.92840554042539
A0A654G0X9	(thale cress) hypothetical protein	4.63570738163864
Q8LA13	DEAD-box ATP-dependent RNA helicase 11	4.28434865158988
O65396	Aminomethyltransferase, mitochondrial	4.07944778868964
A0A7G2F4Y1	(thale cress) hypothetical protein	4.0751098301125
A0A7G2E7L8	(thale cress) hypothetical protein	3.97297184218234
A0A178U6W0	BXL1	3.9247761041483
A0A654EA62	ATP citrate synthase	3.78290364743306
A0A178WLU9	PMEAMT	3.74047331617429
A0A5S9Y3X6	Uncharacterized protein	3.52227914492748
A0A7G2EHP4	Citrate synthase	3.48395995576783
A0A7G2E0Q6	(thale cress) hypothetical protein	3.47192109849934
A0A5S9XHW7	Acid phosphatase	3.46010866456963
A0A178UTZ9	TAF15b	3.40914291941372
A0A178WBS0	(thale cress) hypothetical protein	3.35018979405086
A0A178UAN3	Succinate dehydrogenase [ubiquinone]	3.22519496842387

	flavoprotein subunit, mitochondrial	
Q8LCA1	Protein CURVATURE THYLAKOID 1B, chloroplastic	3.22251958419975
A0A654FYA1	ACT domain-containing protein	3.1819599875211
A0A178V1L3	(thale cress) hypothetical protein	3.13591900871491
A0A6B9JEL0	Small RNA binding protein 1	3.00421035251383
A0A7G2DW29	(thale cress) hypothetical protein	2.89298585179248
W8PUR4	Alpha-1,4 glucan phosphorylase (Fragment)	2.85465730441807
A0A7G2FBM7	AP-2 complex subunit alpha	2.72910623014228
A0A178WD57	Ribulose bisphosphate carboxylase small subunit	2.63591883949228
B9DFJ8	AT4G39260 protein	2.57549674447785
O03042	Ribulose bisphosphate carboxylase large chain	2.53559494962377
A0A178WG36	(thale cress) hypothetical protein	2.53382153232599
A7KNI1	NADH dehydrogenase subunit 7 (Fragment)	2.51234653403009
Q9LIK1	ADP-ribosylation factor C1	2.38068889969428
A0A654G2F8	5-methyltetrahydropteroyltriglutamate--homocysteine S-methyltransferase	2.33811384018527
P38418	Lipoxygenase 2, chloroplastic	2.27497748655412
A0A178UXQ6	Nucleoside diphosphate kinase	2.21218558505982
A0A384LEC5	(thale cress) hypothetical protein	2.18706193863247
A0A1P8BCV2	Acetyl-coenzyme A synthetase	
A0A7G2DZF0	(thale cress) hypothetical protein	
A0A654FIP0	Galactinol--sucrose galactosyltransferase	
A0A7G2ELB8	(thale cress) hypothetical protein	
A0A654EQK1	Uncharacterized protein	
A0A7G2E0M6	(thale cress) hypothetical protein	
A0A7G2E075	(thale cress) hypothetical protein	
A0A178WEL2	(thale cress) hypothetical protein	
A0A7G2DYR6	(thale cress) hypothetical protein	

Table S3 The IP-WHIRLY1 DEPs (P<0.05) in IP-DMMIB/IP-L or IP-D.

Protein ID	Protein Name	IP-DBMID/IP-L
A0A5S9WNH0	AAA domain-containing protein	0.48576307609655
A0A7G2E4J1	Protein-lysine N-methyltransferase AT9943_LOCUS4789	0.209093107617896
A0A5S9Y3J7	Uncharacterized protein	0.16327832543616
Q9SFF5	F10A16.20 protein	0.162091125421815
A0A7G2DWB6	Adenine phosphoribosyltransferase	0.125476765853375
A0A178W6W4	Alanine--tRNA ligase	0.122741372625048
Q9LYA9	Chloroplast stem-loop binding protein of 41 kDa a, chloroplastic	0.111351401133633
A0A5S9X6E0	RNA helicase	0.0934618420554405
Q9LVJ1	Subtilisin-like protease SBT1.4	0.0826405152224824
Q058Q9	At1g32500	0.0669099461178341
A0A178VBG6	40S ribosomal protein S3a	0.0656537958115183
A0A1P8BDK6	ADP-ribosylation factor A1B	0.0454515641065034
A0A7G2ET00	Glyceraldehyde-3-phosphate dehydrogenase	0.0451913554537461
A0A178UBZ3	CaS	0.0427569985703085
A0A7G2F1A7	(thale cress) hypothetical protein	0.0334302318204312
Q8L4Q6	Neurofilament light protein	0.0333745833143066
A0A178WD57	Ribulose bisphosphate carboxylase small subunit	0.0313421496904896
Q96292	Actin-2	0.0253439804983476
Q8LCW6	Similar to late embryogenesis abundant proteins	0.0218195210395105
A0A5S9WIZ3	(thale cress) hypothetical protein	0.00746611548372531
Q8LPR9	Protein TIC110, chloroplastic	0.00567310021804086
P55737	Heat shock protein 90-2	0.00149516204790982
A0A654FL84	CS domain-containing protein	0.000734578037716042
A0A7G2EMC9	Acetyltransferase component of pyruvate dehydrogenase complex	0.000331002691404684
A1L4Y1	ATPase GET3B	
A0A178W3D8	(thale cress) hypothetical protein	
A0A654G681	Cysteinyl-tRNA synthetase	
O04485	Leucyl-tRNA synthetase	
A0A654EWQ2	Uncharacterized protein	
A0A5S9WTE4	Uncharacterized protein	
A0A178WGD6	(thale cress) hypothetical protein	
F4IG09	Subtilisin-like protease SBT4.1	
F4J5M9	Clathrin light chain 3	
A0A7G2EIB7	Inositol hexakisphosphate and diphosphoinositol-pentakisphosphate kinase	

A0A5S9XAD8	HATPase_c domain-containing protein	
A0A178V0W7	Importin subunit alpha	
A0A7G2EX09	Isoleucyl-tRNA synthetase	
A0A1P8B4Q3	Ubiquitin-like superfamily protein	
A0A7G2F1T1	(thale cress) hypothetical protein	
A0A7G2FCC7	(thale cress) hypothetical protein	
A0A178W962	D-3-phosphoglycerate dehydrogenase	
A0A654F0F3	Eukaryotic translation initiation factor 3 subunit F	
Q8LBW1	Putative lectin	
A0A1P8ARS7	CCT-epsilon	
B9DHI9	AT1G09640 protein	
A0A178W448	(thale cress) hypothetical protein	
A0A5S9Y814	Threonyl-tRNA synthetase	
Q8VYP7	Putative Sar1 GTP binding protein	
A0A178VL71	(thale cress) hypothetical protein	
A0A5S9X8K6	Chromo domain-containing protein	
A0A178VX04	(thale cress) hypothetical protein	
A0A178VS42	MORF2	
A0A178VC46	Uncharacterized protein	
O22914	Calvin cycle protein CP12-1, chloroplastic	
O23140	AP-2 complex subunit mu	
A0A178V430	(thale cress) hypothetical protein	
A0A654FNZ0	Uncharacterized protein	
A0A7G2F2G9	Sulfate adenylyltransferase	
A0A654FPF3	Ferredoxin	
A0A7G2F097	Ribosomal protein L15	
W8PUL2	Glycosyltransferase (Fragment)	
A0A1P8BBD5	Aspartokinase	
A0A178WA13	Proteasome subunit alpha type	
A0A7G2EY26	Proteasome subunit beta	
A0A178WCQ3	Proteasome subunit beta	
A0A654FYV3	MPN domain-containing protein	
Q56ZC8	Hydroxyacylglutathione hydrolase cytoplasmic (Fragment)	
A0A7G2F3V6	40S ribosomal protein S6	
A0A654F1E6	Uncharacterized protein	
A0A178W2K0	Protein disulfide-isomerase	
A0A178WN90	(thale cress) hypothetical protein	
A0A654F8W6	26S proteasome non-ATPase regulatory subunit 1 homolog	
A0A654F735	Pectinesterase	
A0A654FH19	Eukaryotic translation initiation factor 3 subunit C	
A0A654ENJ4	GDP-L-fucose synthase	

B9DHI2	AT4G21210 protein	
A0A178V235	(thale cress) hypothetical protein	
O49675	Probable carotenoid cleavage dioxygenase 4, chloroplastic	
Q2V4Q4	AT1G07320 protein	
A0A1P8B3J7	Calmodulin-binding family protein	
A8MQM3	Ribose-phosphate diphosphokinase	
O65282	20 kDa chaperonin, chloroplastic	
A0A654GEW7	Uncharacterized protein	
A0A7G2DT19	(thale cress) hypothetical protein	
A0A7G2DT14	(thale cress) hypothetical protein	
O65572	Carotenoid 9,10(9,10)-cleavage dioxygenase 1	
A0A178V385	Fructose-bisphosphate aldolase	
A0A5S9XYN6	Uncharacterized protein	
A0A178WCC0	(thale cress) hypothetical protein	
A0A7G2EII3	(thale cress) hypothetical protein	
A0A654FCH5	Uncharacterized protein	
A0A7G2EHQ0	Phosphomannomutase	
A0A7G2EGL3	(thale cress) hypothetical protein	
C0Z300	AT2G37660 protein	
A0A1P8B070	Hydroxymethylglutaryl-CoA lyase	
A0A178VJ65	Proteasome subunit alpha type	
A0A654F8R2	Proteasome subunit alpha type	
A0A178VXA5	(thale cress) hypothetical protein	
A0A178V663	(thale cress) hypothetical protein	
A0A178VWV6	(thale cress) hypothetical protein	
A0A7G2ECH1	(thale cress) hypothetical protein	
A0A654EW41	Succinate--CoA ligase [ADP-forming] subunit beta, mitochondrial	
A0A5S9Y7Y8	Serine/threonine-protein phosphatase	
A0A178WBP5	(thale cress) hypothetical protein	
Q1H5F3	At3g43810	
P11139	Tubulin alpha-1 chain	
A0A5S9WVV8	30S ribosomal protein S17, chloroplastic	
A0A178VH97	(thale cress) hypothetical protein	
A0A5S9XP07	S-adenosylmethionine synthase	
A0A7G2EUY8	Acetolactate synthase	
A0A654FHK1	Protein kinase domain-containing protein	
Q0WLB0	Xyloglucan endotransglucosylase/hydrolase	
A0A178VT47	Phosphopyruvate hydratase	
A0A654FGI2	D-fructose-1,6-bisphosphate 1-phosphohydrolase	
A0A178VBQ7	50S ribosomal protein L9, chloroplastic	
P25873	50S ribosomal protein L15, chloroplastic	
C0Z3A1	AT2G30620 protein	

P27521	Chlorophyll a-b binding protein 4, chloroplastic	
A0A654FE88	CCT-alpha	
A0A178UXP7	Tubulin alpha chain	
A0A178VS27	Tubulin beta chain	
A0A178UKM5	Tubulin beta chain	
P29517	Tubulin beta-9 chain	
Q0WL48	Uncharacterized protein At1g20440	
A0A654F7V9	TCTP domain-containing protein	
Q8H7B4	Glucan endo-1,3-beta-D-glucosidase (Fragment)	
Q5PNZ9	At1g22780	
P37107	Signal recognition particle 54 kDa protein, chloroplastic	
A0A178U7L7	TGG1	
A0A5S9XFR2	SAICAR synthetase	
P38420	DNA-directed RNA polymerase II subunit 2	
A0A5S9XKB3	TRASH domain-containing protein	
A0A178UXQ6	Nucleoside diphosphate kinase	
P40940	ADP-ribosylation factor 3	
A0A178UQ77	GTP-binding nuclear protein	
P42036	40S ribosomal protein S14-3	
A0A5S9XQK2	14_3_3 domain-containing protein	
A0A178UA42	(thale cress) hypothetical protein	
F4K015	Dynamin-like protein	
A0A178WAZ3	Plastocyanin	
A0A178UPD3	(thale cress) hypothetical protein	
A0A178V326	(thale cress) hypothetical protein	
F4JD01	Proteasome subunit beta	
F4HST2	Dehydrin family protein	
A0A384KNV6	(thale cress) hypothetical protein	
A0A654GDN2	Glutamate-1-semialdehyde 2,1-aminomutase	
A0A654EJU2	Glutamyl-tRNA reductase	
A0A178UA36	SRK2G	
A0A5S9TI57	(thale cress) hypothetical protein	
A0A654FDY3	CN hydrolase domain-containing protein	
Q94BW0	At1g35160/T32G9_30	
F4JTH0	Aspartate aminotransferase	
A0A654FS26	Gamma-ECS	
A0A178V0A3	Glutathione transferase	
A0A7G2EY35	Cysteine synthase	
Q0WW95	Cysteine synthase	
P48348	14-3-3-like protein GF14 kappa	
A0A178UNB4	(thale cress) hypothetical protein	
A0A178VP20	(thale cress) hypothetical protein	

A0A178UJU5	(thale cress) hypothetical protein	
Q7G923	60S ribosomal protein L27A; 71521-71081	
F4KDU5	Ribosomal protein L4/L1 family	
A0A178UUL5	Ribosomal protein L19	
A8MSA9	Signal recognition particle 54 kDa protein	
F4I3L1	Phosphoglycerate kinase	
A0A178VF24	(thale cress) hypothetical protein	
A8MS28	Ribosomal L27e protein family	
P51427	40S ribosomal protein S5-2	
A0A178U8T0	Hsp81.3	
A0A178VXY6	Glutathione peroxidase	
Q541W9	Uncharacterized protein At3g12110/T21B14_108	
A0A7G2F0R0	Protein-methionine-S-oxide reductase	
A0A7G2F961	(thale cress) hypothetical protein	
A0A7G2F639	Glucose-1-phosphate adenylyltransferase	
A0A514YJ21	ATP synthase subunit b, chloroplastic	
A0A178VY74	Photosystem I P700 chlorophyll a apoprotein A1	
P56767	Photosystem I P700 chlorophyll a apoprotein A2	
A0A7G2FNE9	Cytochrome f	
A0A1B1W4W5	ATP-dependent Clp protease proteolytic subunit	
P56773	Cytochrome b6	
A0A514YJW6	50S ribosomal protein L16, chloroplastic	
A0A1B1W4W8	50S ribosomal protein L20, chloroplastic	
A0A1B1W4X3	50S ribosomal protein L33, chloroplastic	
A0A1B1W4W9	30S ribosomal protein S18, chloroplastic	
A0A1B1W4X5	30S ribosomal protein S19, chloroplastic	
A0A178W1V2	Ribosomal protein	
A0A178VVS2	(thale cress) hypothetical protein	
P60040	60S ribosomal protein L7-2	
A0A1B1W4W0	Photosystem II reaction center protein L	
A0A178VIZ0	PIP1A	
Q6KGZ1	30S ribosomal protein S7, chloroplastic	
A0A1B1W4Y9	50S ribosomal protein L32, chloroplastic	
A0A178V984	(thale cress) hypothetical protein	
A0A654FZJ9	ATP synthase subunit beta	
A0A178V4V1	(thale cress) hypothetical protein	
A0A654ELQ9	Thioredoxin domain-containing protein	
A8MS49	Phototropin 2	
A7KNI1	NADH dehydrogenase subunit 7 (Fragment)	

B9DHX4	Malate dehydrogenase (Fragment)	
A0A7G2E3W0	(thale cress) hypothetical protein	
Q39209	RNA binding protein (Fragment)	
B9DFR9	AT2G45960 protein	
Q06738	Low-temperature-induced 78 kDa protein	
Q93ZB4	Phytoene dehydrogenase	
B0FV99	At1g04270 (Fragment)	
A0A178UH82	RD22	
A0A7G2DWN9	(thale cress) hypothetical protein	
A0A7G2E8R3	(thale cress) hypothetical protein	
A0A654EHJ3	Serine/threonine-protein phosphatase 2A 55 kDa regulatory subunit B	
A0A7G2F8C1	(thale cress) hypothetical protein	
Q0WVZ3	Eukaryotic translation initiation factor 3 subunit I	
A0A178VM60	Zeta-carotene desaturase	
A0A178VIW5	PR 65	
A0A178UG43	BIP2	
A0A7G2FBG2	Ferritin	
A0A7G2F7N1	(thale cress) hypothetical protein	
A0A5S9YHP7	(thale cress) hypothetical protein	
A0A178VVN9	(thale cress) hypothetical protein	
A0A178VNK7	PSBTN	
A0A178WD95	SKP1-like protein	
A0A178UBS9	40S ribosomal protein S21	
Q41188	Cold shock protein 2	
A0A178UYN8	(thale cress) hypothetical protein	
A8MQR4	60S acidic ribosomal protein P0	
A0A178UZT1	40S ribosomal protein S3a	
F4JWM1	Ribosomal protein S5 domain 2-like superfamily protein	
Q42560	Aconitate hydratase 1	
A0A5S9XKR6	OMPdecase	
A0A7G2DWI3	Carbamoyl-phosphate synthase (glutamine-hydrolyzing)	
Q0WSS0	Uncharacterized protein At1g57860	
A0A5S9XKG3	(thale cress) hypothetical protein	
A0A654G692	Glutamine synthetase	
A0A654GBM6	Aldo_ket_red domain-containing protein	
Q0WLJ0	Peroxisomal-3-keto-acyl-CoA thiolase 1	
A0A7G2DXI6	Thiamine-phosphate pyrophosphorylase	
A0A654G5U7	RRM domain-containing protein	
A0A7G2E2R1	Proteasome subunit beta	
A0A178UH95	(thale cress) hypothetical protein	
Q29PY2	At5g12110	
A0A5S9TIS9	ATP phosphoribosyltransferase	

Q8GWE0	Pentatricopeptide repeat-containing protein At4g16390, chloroplastic	
A0A5S9Y1A3	Rhodanese domain-containing protein	
Q8GYA6	26S proteasome non-ATPase regulatory subunit 13 homolog B	
A0A7G2E6D2	HECT-type E3 ubiquitin transferase	
A0A7G2F5W0	Dihydrolipoyllysine-residue succinyltransferase	
A0A178VXZ7	(thale cress) hypothetical protein	
A0A178VQL0	(thale cress) hypothetical protein	
A0A654F363	Uncharacterized protein	
A0A178WHW4	(thale cress) hypothetical protein	
A0A178V304	Coatomer subunit zeta	
A0A178UBK9	Cytosolic Fe-S cluster assembly factor NBP35	
A0A7G2DT04	Pyruvate dehydrogenase E1 component subunit alpha	
A0A5S9XWB6	Usp domain-containing protein	
A0A654GDZ1	Uncharacterized protein	
Q1H5B6	At5g42970	
B7T504	Putative ATP-dependent Clp protease proteolytic subunit (Fragment)	
F4JXE7	RNA-binding (RRM/RBD/RNP motifs) family protein	
F4K180	RNA helicase	
A0A7G2E238	(thale cress) hypothetical protein	
A0A178V3F1	Serine/threonine-protein phosphatase	
G1JSI6	At1g59359	
F6M3L4	Pyridoxine biosynthesis protein	
A0A178V785	ATP-dependent Clp protease proteolytic subunit	
A0A654FTI9	GrpE protein homolog	
A0A654FEW2	Uncharacterized protein	
A0A7G2EL76	(thale cress) hypothetical protein	
A0A178US36	40S ribosomal protein S24	
A0A178VE56	60S ribosomal protein L27	
A0A178USJ2	ACHT2	
A0A178WHE0	Uncharacterized protein	
Q8LD03	40S ribosomal protein S7-3	
A0A178V2B3	Proteasome subunit beta	
A0A7G2E784	(thale cress) hypothetical protein	
A0A7G2F3R2	Xyloglucan endotransglucosylase/hydrolase	
A0A5S9X9I0	AMP-binding domain-containing protein	
A0A654FLY8	Uncharacterized protein	
A0A7G2EP20	(thale cress) hypothetical protein	
A0A178V6Q6	(thale cress) hypothetical protein	
A0A5S9XD62	Thioredoxin domain-containing protein	
Q8VY91	30S ribosomal protein S6 alpha, chloroplastic	
A0A384KKR2	(thale cress) hypothetical protein	

A0A7G2E2S1	(thale cress) hypothetical protein	
A0A178U8I7	Era-type G domain-containing protein	
Q8VZC7	Probable disease resistance protein At5g45510	
A0A178UB34	FUS11	
A0A178V1W8	(thale cress) hypothetical protein	
A0A178UCD3	40S ribosomal protein S8	
A0A654FP28	Uncharacterized protein	
B9DGK9	Coatomer subunit delta	
A0A654FRT4	STI1 domain-containing protein	
A0A1P8B108	N-acetyl-glutamate semialdehyde dehydrogenase	
A0A654EX31	Uncharacterized protein	
A0A178UVE7	(thale cress) hypothetical protein	
A0A178V700	(thale cress) hypothetical protein	
A0A178UKW4	(thale cress) hypothetical protein	
A0A178UL10	CCT-beta	
A0A178UAL4	(thale cress) hypothetical protein	
A0A5S9Y6C6	HTH La-type RNA-binding domain-containing protein	
A0A7G2DZF1	(thale cress) hypothetical protein	
A0A178W6I0	SNF4	
A0A178UB52	Isocitrate dehydrogenase [NAD] subunit, mitochondrial	
A0A654ETK5	(thale cress) hypothetical protein	
A0A178VHG0	Glutaredoxin-dependent peroxiredoxin	
Q8L8N9	Spore coat protein-like protein	
A0A7G2DQW8	(thale cress) hypothetical protein	
A0A654G0X9	(thale cress) hypothetical protein	
Q3ECU3	AT hook motif-containing protein	
A0A7G2FM21	(thale cress) hypothetical protein	
A0A5S9WRP0	(thale cress) hypothetical protein	
A0A178UWP5	3-isopropylmalate dehydratase	
F4J1V2	DNAJ homologue 3	
A0A5S9U051	Uncharacterized protein	
A0A5S9XZ93	Uncharacterized protein	
F4JQD8	Translation initiation factor 3 protein	
B3H5Y8	Glycine cleavage system P protein	
A0A5S9YIS7	Uncharacterized protein	
A0A7G2FAM5	DNA gyrase subunit B	
A0A5S9Y5Z9	Cytochrome b-c1 complex subunit Rieske, mitochondrial	
F4HVW0	Proteasome component (PCI) domain protein	
A0A7G2ERG6	(thale cress) hypothetical protein	
A0A178W0Z6	ATP synthase subunit gamma	
Q96262	Plasma membrane-associated cation-binding protein 1	

A0A178VHS3	Importin subunit alpha	
A0A5S9XG25	(thale cress) hypothetical protein	
Q0WUH6	Catalase	
A0A654G7Z5	WPP domain-containing protein	
A0A178WLT0	Eukaryotic translation initiation factor 5A	
A0A178UIY8	Uncharacterized protein	
Q8LEA5	Thioredoxin-dependent peroxiredoxin	
Q9C5Z2	Eukaryotic translation initiation factor 3 subunit H	
A0A178WI45	(thale cress) hypothetical protein	
A0A5S9WU24	Uncharacterized protein	
A0A5S9WLM8	RanBD1 domain-containing protein	
Q9C865	SH3 domain-containing protein 1	
A0A654EQA9	Ribosomal_L6e_N domain-containing protein	
A0A178WG16	3-oxoacyl-[acyl-carrier-protein] synthase	
A0A178W4H7	(thale cress) hypothetical protein	
A0A178WMJ1	Nascent polypeptide-associated complex subunit beta	
Q0WRH6	60S ribosomal protein L22-like	
F4JV93	PHD finger family protein / bromo-adjacent homology (BAH) domain-containing protein	
A0A5S9Y905	60S ribosomal protein L13	
A0A654G6Z8	6-phosphogluconate dehydrogenase, decarboxylating	
A0A1P8B9L0	Methylthioalkylmalate synthase 1	
Q8L7R7	AT4g37510/F6G17_160	
A0A178U6W0	BXL1	
A0A178UGP2	Uncharacterized protein	
A0A7G2FG07	(thale cress) hypothetical protein	
A0A7G2FKQ2	(thale cress) hypothetical protein	
Q9FJA6	40S ribosomal protein S3-3	
B9DH43	AT5G65220 protein	
Q9FKA5	Uncharacterized protein At5g39570	
A0A178UGQ5	NEET	
Q8LEF5	50S ribosomal protein L27	
A0A178UNS2	Small ubiquitin-related modifier	
A0A7G2FK60	Dihydrolipoyllysine-residue succinyltransferase	
Q9FLT0	Ribonuclease TUDOR 2	
A0A7G2FGK7	Glucosidase 2 subunit beta	
A0A7G2FHT9	(thale cress) hypothetical protein	
A0A7G2FBK3	RuvB-like helicase	
A0A178UMU4	(thale cress) hypothetical protein	
A0A178UL71	RSW3	
B0ZVS7	NHL3 (Fragment)	

Q67ZX8	40S ribosomal protein S19-like	
A0A178UNN6	Uncharacterized protein	
A0A7G2F8Y2	Ubiquitinyl hydrolase 1	
Q8GUG4	Uncharacterized protein	
A0A654G1P3	Peptide deformylase	
A0A178VK18	6-phosphogluconate dehydrogenase, decarboxylating	
A0A7G2E323	(thale cress) hypothetical protein	
Q9FZ76	60S ribosomal protein L6-1	
A0A178VH27	(thale cress) hypothetical protein	
A0A654G1T5	Uncharacterized protein	
Q0WW72	60S ribosomal Protein L12-like	
A0A7G2EIJ8	tRNA (guanine(26)-N(2))-dimethyltransferase	
Q93ZK4	AT3g26420/F20C19_14	
F4JDC2	Evolutionarily conserved C-terminal region 2	
A0A7G2ERE5	(thale cress) hypothetical protein	
Q9LK61	30S ribosomal protein S10, chloroplastic	
Q9LKR8	Rubisco accumulation factor 1.1, chloroplastic	
A0A5S9VBJ8	Small nuclear ribonucleoprotein Sm D3	
A0A178WH64	(thale cress) hypothetical protein	
A0A654E993	Uncharacterized protein	
A0A178WBT3	EDA10	
A0A7G2DV36	(thale cress) hypothetical protein	
A0A178W322	Alkaline/neutral invertase	
Q9LQR8	26S proteasome non-ATPase regulatory subunit 3 homolog B	
Q9LS02	Allene oxide cyclase 2, chloroplastic	
A0A178UPY9	(thale cress) hypothetical protein	
A0A654F8F2	(thale cress) hypothetical protein	
A0A178VBY8	(thale cress) hypothetical protein	
A0A5S9XDV3	Uncharacterized protein	
A0A5S9XC85	(thale cress) hypothetical protein	
A0A654GA42	Uncharacterized protein	
A0A5S9XES0	DUF2470 domain-containing protein	
A0A5S9XD98	T-complex protein 1 subunit delta	
A0A654FHE8	Uncharacterized protein	
A0A5S9YCP5	Uncharacterized protein	
A0A178U7Z1	Amylomaltase	
A0A7G2FKL7	(thale cress) hypothetical protein	
A0A178UG88	(thale cress) hypothetical protein	
A0A7G2FH56	(thale cress) hypothetical protein	
A0A178UPS5	Aminotran_1_2 domain-containing protein	
B3H4M0	Post-illumination chlorophyll fluorescence increase	
A0A178UUQ0	(thale cress) hypothetical protein	
Q9LY74	2-methyl-6-phytyl-1,4-hydroquinone methyltransferase, chloroplastic	

A0A5S9Y0T5	GST N-terminal domain-containing protein	
A0A5S9Y2X6	60S ribosomal protein L36	
A0A654FY85	Assimilatory sulfite reductase (ferredoxin)	
A0A7G2F758	(thale cress) hypothetical protein	
A0A178V9A8	60S ribosomal protein L7a	
Q9LZK5	DnaJ protein ERDJ3B	
A0A178VF73	(thale cress) hypothetical protein	
A0A654FTY1	Ribosomal_L28e domain-containing protein	
Q9M0N1	Plant UBX domain-containing protein 10	
A0A178UTB9	Vesicle-fusing ATPase	
F4J0X9	Homocysteine methyltransferase 2	
A0A178VG46	40S ribosomal protein S21	
A0A178VA59	60S ribosomal protein L36	
A0A6B7JDQ1	Proliferating cell nuclear antigen	
A0A178VB48	40S ribosomal protein S7	
A0A654F3F3	Uncharacterized protein	
A0A178VCA4	AtACBP4	
A0A178WA97	SDC1	
A0A7G2E247	(thale cress) hypothetical protein	
A0A7G2E0Q6	(thale cress) hypothetical protein	
A0A654EGJ2	Uncharacterized protein	
A0A178W5Z5	Protein disulfide-isomerase	
A0A654F5U8	Ferritin	
A0A654ETC9	Chlorophyll a-b binding protein, chloroplastic	
A0A7G2FLZ2	(thale cress) hypothetical protein	
A0A7G2DZB5	(thale cress) hypothetical protein	
Q9S834	ATP-dependent Clp protease proteolytic subunit 5, chloroplastic	
Q8GXQ8	Putative inosine-5-monophosphate dehydrogenase	
A0A178WF69	Obg-like ATPase 1	
F4JN17	Ankyrin repeat-containing protein 2	
A0A5S9Y678	(thale cress) hypothetical protein	
A0A384KZD9	RPR2	
Q9SE60	Methylenetetrahydrofolate reductase 1	
A0A7G2ELV3	(thale cress) hypothetical protein	
A0A178VGX5	CCT-eta	
A0A7G2EH20	(thale cress) hypothetical protein	
Q9SFH9	Delta-aminolevulinic acid dehydratase 1, chloroplastic	
A0A7G2EKN8	(thale cress) hypothetical protein	
A0A7G2EF58	(thale cress) hypothetical protein	
A0A178WCP0	AXS2	
A0A178WH62	RPN12a	
Q9SHI1	Translation initiation factor IF-2, chloroplastic	
A0A654EW86	(thale cress) hypothetical protein	

F4IHJ8	40S ribosomal protein S25	
A0A097PNI8	ABH1 (Fragment)	
A0A178VYF4	(thale cress) hypothetical protein	
A0A5S9X4B0	Sucrose-phosphatase	
A0A7G2EHP4	Citrate synthase	
Q9SJI7	Phospholipase A1-IIdelta	
A0A7G2DVD0	(thale cress) hypothetical protein	
A0A654F8M7	Protein kinase domain-containing protein	
A8MRE8	Triosephosphate isomerase	
Q9SKU1	Clathrin light chain 1	
B9DGN3	AT2G27710 protein	
A0A654EU45	Isocitrate dehydrogenase [NADP]	
A0A178U9W2	(thale cress) hypothetical protein	
Q9SN73	Protein BUNDLE SHEATH DEFECTIVE 2, chloroplastic	
A0A654FEK4	Malate dehydrogenase	
A0A178VDJ4	UDP-glucuronate decarboxylase	
A0A178USI0	Uncharacterized protein	
A0A178V7M0	(thale cress) hypothetical protein	
A0A178VCP4	(thale cress) hypothetical protein	
A0A654FGH8	Uncharacterized protein	
Q9SRZ6	Cytosolic isocitrate dehydrogenase [NADP]	
A0A1I9LMY7	40S ribosomal protein S24	
Q9SSA5	Peptidyl-prolyl cis-trans isomerase CYP38, chloroplastic	
A0A178V791	Vacuolar protein sorting-associated protein 29	
F4JP99	Tyrosine transaminase family protein	
A0A178W0C4	Serine/threonine-protein phosphatase	
Q0WWC5	Uncharacterized protein At1g48350	
A0A178WJK9	ATPI4K ALPHA	
B9DF86	Lipoyl(octanoyl) transferase (Fragment)	
A0A7G2FEQ0	Cysteine synthase	
A0A178V1I1	Small nuclear ribonucleoprotein Sm D1	
A0A178W5Y6	Chlorophyll a-b binding protein, chloroplastic	
A0A654FV66	Eukaryotic translation initiation factor 3 subunit K	
A0A654FU27	Elongation factor Ts, mitochondrial	
A0A178UYK0	VIP3	
A8MQP6	Nascent polypeptide-associated complex (NAC), alpha subunit family protein	
Q9XF88	Chlorophyll a-b binding protein CP29.2, chloroplastic	
A0A7G2E1Y9	(thale cress) hypothetical protein	
Q9XFS9	1-deoxy-D-xylulose 5-phosphate reductoisomerase, chloroplastic	
Q0WWD3	Vacuolar-type H+-ATPase (V-ATPase) subunit D	

A0A654EGZ5	(thale cress) hypothetical protein	
A0A178W7K9	MBD10	
A0A178VQU8	(thale cress) hypothetical protein	
B9DFC0	AT4G08870 protein	
A0A654FLS1	Thioredoxin domain-containing protein	
A0A7G2EV07	(thale cress) hypothetical protein	
F4INA0	Nicotinate-nucleotide pyrophosphorylase [carboxylating]	
A0A7G2EBV3	UDP-glucuronate decarboxylase	
A0A7G2FEV2	(thale cress) hypothetical protein	
Q94K56	GrpE protein homolog	
Q8LD96	Nodulin-like protein	
A0A178UM83	Eukaryotic translation initiation factor 3 subunit L	
A0A1P8BCE3	Octicosapeptide/Phox/Bem1p family protein	
A0A7G2FDR7	(thale cress) hypothetical protein	
Q8L745	AT5g42220/K5J14_2	
Q93XY1	At5g53620	
A0A178UCE0	(thale cress) hypothetical protein	
Q9FXM2	DC 1.2 homolog (Fragment)	
A0A7G2F9F2	(thale cress) hypothetical protein	
Q93ZC2	ARM repeat superfamily protein	
Q9FM97	Pyruvate kinase	
Q9FJZ7	AT5G66420 protein	
A0A7G2FAP9	(thale cress) hypothetical protein	
Q9FIS4	ATP-dependent Clp protease ATP-binding subunit	
A0A1P8BG25	Glycosyl hydrolase family protein	
Q9FM47	RNA-binding (RRM/RBD/RNP motifs) family protein	
A0A654G6U7	Uncharacterized protein	
F4K0V9	Octanoyltransferase	
A0A7G2FNH5	(thale cress) hypothetical protein	
Q93VS8	ARM repeat superfamily protein	
Q9SD78	Furry	
Q9LX13	(3R)-hydroxymyristoyl-[acyl-carrier-protein] dehydratase	
Q94K88	Tetratricopeptide repeat (TPR)-like superfamily protein	
A0A178UT81	Carbonic anhydrase	
Q8L816	Uncharacterized protein At4g09520	
Q9SMQ9	DnaJ-like protein	
Q9SST2	At4g09620	
A0A654FVA8	ENDO3c domain-containing protein	
Q9M0Y0	Uncharacterized protein AT4g05150	
F4JEJ3	Metallo-hydrolase/oxidoreductase superfamily protein	
Q9LX44	FBD-like domain family protein	
Q9SD66	Thylakoid soluble phosphoprotein	

Q42338	AT3G48140 protein	
A0A654F8A7	H15 domain-containing protein	
Q8GWM3	Uncharacterized protein At3g24506/MOB24.5	
A0A654FGL7	Eukaryotic translation initiation factor 3 subunit G	
Q9LF93	Pyruvate kinase	
A0A7G2EUV4	Small nuclear ribonucleoprotein Sm D2	
Q9SVD1	At3g52500	
Q9M1Q0	RING-type E3 ubiquitin transferase	
F4J6A8	DnaJ and Myb-like DNA-binding domain-containing protein	
Q9SIJ8	Adenine nucleotide alpha hydrolases-like superfamily protein	
Q8LBA5	Putative monooxygenase	
F4ISU2	Early endosome antigen	
O80897	At2g32600/T26B15.16	
Q9SIE3	(3R)-hydroxymyristoyl-[acyl-carrier-protein] dehydratase	
A0A7G2EDL4	(thale cress) hypothetical protein	
Q9CA53	Uncharacterized protein F1M20.32	
Q9SHG8	At1g17100	
Q9LPS0	F11F12.19 protein	
Q9C9G5	Adenine nucleotide alpha hydrolases-like superfamily protein	
Q9C685	At1g51100	
Q9C9I7	Rieske (2Fe-2S) domain-containing protein	
A0A1P8ARI3	RNA polymerase II degradation factor-like protein (DUF1296)	
A0A654EJB7	DUF2431 domain-containing protein	
A0A178WBF4	DNL-type domain-containing protein	
Q9C7S3	Inner membrane localized protein	
Q4V3D1	At1g48620	
Q9FE18	Peptidyl-prolyl cis-trans isomerase	
Q8VZ31	Tetratricopeptide repeat (TPR)-like superfamily protein	
A0A7G2E766	(thale cress) hypothetical protein	
A0A5S9VBT6	Uncharacterized protein	
Q9SSG7	F25A4.12 protein	
A0A5S9WTK4	ANAPC4_WD40 domain-containing protein	
Q0WUH5	Uncharacterized protein At1g19691	
Q680H0	mRNA, clone: RAFL22-54-C15	
B9DGR7	AT1G49750 protein (Fragment)	
O81909	T7I23.15 protein	
A0A1I9LLZ2	SIT4 phosphatase-associated family protein	
A0A1I9LMI8	Transmembrane protein	
A0A1I9LQL1	GDSL-like lipase/acylhydrolase superfamily protein	
A0A1P8ART2	Molecular chaperone Hsp40/DnaJ family protein	

F4HSW8	GYF domain-containing protein	
Q9LPP9	F15H18.21	
A0A1P8B9Q7	Transcription regulator NOT2/NOT3/NOT5 family protein	
Q8LAP5	Protein-synthesizing GTPase	
Q9FFP6	Pyruvate kinase	
Q8W4A0	Eukaryotic translation initiation factor 3 subunit M	
Q94JV2	AT3g27050/MOJ10_14	
Q9LIK1	ADP-ribosylation factor C1	
Q8LDN2	Ankyrin repeat family protein	
Q8GRX2	Eukaryotic translation initiation factor 3 subunit J	
Q9C6U3	Uncharacterized protein T8G24.2 (Fragment)	
Q93VK7	AT5g14910/F2G14_30	
Q9M0V5	At4g05400	
Q8L7C1	Expressed protein	
O82762	F17H15.1/F17H15.1	
A0A7G2F273	(thale cress) hypothetical protein	
A0A5S9Y7L0	MADS-box domain-containing protein	
A0A654E9D1	Uncharacterized protein	
A8MQJ8	Glucose-induced degradation-like protein	
Q9SFV3	At3g07230	
F4JE18	Dentin sialophosphoprotein-like protein	
A0A7G2F5E9	Isocitrate dehydrogenase [NAD] subunit, mitochondrial	
A0A7G2F9S0	(thale cress) hypothetical protein	
Q8GX17	Ankyrin repeat family protein	
Q6XWB5	Resistance protein Ei2-2 (Fragment)	
Q9FVP6	3-phosphoshikimate 1-carboxyvinyltransferase	
Q9LMR1	At1g15730/F7H2_7	
Q9M1F5	Copia-like polyprotein	
Q9STT3	Aldose 1-epimerase	
Protein ID	Protein Name	IP-DBMIB/IP-D
Q9LV28	Receptor for activated C kinase 1C	0.362198639195628
A0A178V6B0	GDP-mannose 4,6-dehydratase	0.225668655985357
A0A178W6W4	Alanine--tRNA ligase	0.214264302981467
Q9FFQ2	Alcohol dehydrogenase-like protein	0.195424485486172
Q9LRJ9	Cysteine-rich repeat secretory protein 38	0.194965221044769
O82628	V-type proton ATPase subunit G1	0.192046039252199
A0A1P8AV97	ATP-dependent DNA helicase	0.184437287162024
A0A7G2ECL1	Acetohydroxy-acid synthase small subunit	0.183107128035887

Q66GR6	Single-stranded DNA-binding protein WHY3, chloroplastic	0.178724353540311
Q9FNN1	Pyruvate kinase	0.175098548094374
A0A5S9XHW7	Acid phosphatase	0.17293771043771
A0A654EA62	ATP citrate synthase	0.172375861826093
F4IFC5	Threonine--tRNA ligase, chloroplastic/mitochondrial 2	0.156627896808045
Q9STF2	Protein PLASTID TRANSCRIPTIONALLY ACTIVE 16, chloroplastic	0.155303499855378
A0A654E9P6	Uncharacterized protein	0.150183918937836
F4I2F8	NADPH-protochlorophyllide oxidoreductase	0.140389519606522
P52410	3-oxoacyl-[acyl-carrier-protein] synthase I, chloroplastic	0.139156416755063
Q0WWW3	GDP-mannose 3,5-epimerase	0.137437731849497
A0A178UY83	Elongation factor Tu	0.131905722767466
A0A178UEW4	(thale cress) hypothetical protein	0.118136080054358
A0A178WA64	Glyceraldehyde-3-phosphate dehydrogenase	0.11798876669021
A0A178VW16	(thale cress) hypothetical protein	0.11656924193442
F4KFD7	26S proteasome regulatory subunit, putative (RPN5)	0.114726065058174
A0A5S9YDR0	Uncharacterized protein	0.11414134223575
A0A654F4M5	Glutaredoxin-dependent peroxiredoxin	0.108456285436832
Q8GTY0	Elongation factor 1-alpha 4	0.108290435223385
A0A7G2FBM7	AP-2 complex subunit alpha	0.106913722413444
F4K1G7	P-loop containing nucleoside triphosphate hydrolases superfamily protein	0.102753714794935
A0A178UAH2	RPT3	0.101967412990468
P82715	PsbP domain-containing protein 5, chloroplastic	0.0942893136296521
P56757	ATP synthase subunit alpha, chloroplastic	0.0939821608585246
A0A5S9WM95	Glyceraldehyde-3-phosphate dehydrogenase	0.0890167752388024
A0A384L5D6	VIPP1	0.0843705814761197
P49107	Photosystem I reaction center subunit N, chloroplastic	0.0831599871089005
A0A178VCV2	Sulfate adenylyltransferase	0.0827415727953499
A0A178UTZ9	TAF15b	0.0826160337552743
A0A178VZC8	26S proteasome non-ATPase regulatory subunit 2 homolog	0.0818085479682785
A0A654G8S4	ATP-dependent Clp protease proteolytic subunit	0.0794717324377274
A0A1P8AXD8	Tubulin/FtsZ family protein	0.0773942830551271
A0A7G2EJJ6	(thale cress) hypothetical protein	0.0770199037706984
A0A178VI76	(thale cress) hypothetical protein	0.0761034121452326

A0A5S9XVD2	Uncharacterized protein	0.0742675087732999
A0A178W6V9	Phosphoglycerate mutase (2,3-diphosphoglycerate-independent)	0.0735993330584291
F4JBY2	Transketolase	0.0726631797220761
Q8RXP0	Delta-1-pyrroline-5-carboxylate synthase	0.0717793770663984
A0A5S9Y3X6	Uncharacterized protein	0.0695559574040587
Q8LCA1	Protein CURVATURE THYLAKOID 1B, chloroplastic	0.0664943355511599
Q9LYA9	Chloroplast stem-loop binding protein of 41 kDa a, chloroplastic	0.065666829603077
A0A178W3N8	(thale cress) hypothetical protein	0.06462512631929
A0A384KJY5	(thale cress) hypothetical protein	0.0643359554086335
A0A178UL64	(thale cress) hypothetical protein	0.0614384251859059
A0A178W4U1	eIF4A-2	0.0608959084361357
A0A7G2EKV9	(S)-2-hydroxy-acid oxidase	0.0594316581332538
Q0WLF6	V-ATPase 69 kDa subunit	0.0594277125598359
A0A7G2DWB6	Adenine phosphoribosyltransferase	0.0580147405263512
A0A5S9YDC0	Prolyl-tRNA synthetase	0.0572240838212114
A0A178V808	NADPH-protochlorophyllide oxidoreductase	0.0550494907602318
A0A178U9H3	Probable alanine--tRNA ligase, chloroplastic	0.0543732902263119
Q9LVJ1	Subtilisin-like protease SBT1.4	0.0541248972884141
A0A178VIP8	S-adenosylmethionine synthase	0.0535003623844899
A0A7G2FAT4	(thale cress) hypothetical protein	0.052841824295437
Q9SW18	Magnesium protoporphyrin IX methyltransferase, chloroplastic	0.0520026904399239
A0A178VXF8	S-adenosylmethionine synthase	0.0519269480993086
A0A654EP66	1,4-alpha-D-glucan glucanohydrolase	0.0516688015205669
Q9SCY0	Phosphoglucomutase, chloroplastic	0.0508484585323491
A0A7G2ET00	Glyceraldehyde-3-phosphate dehydrogenase	0.0494081635234445
F4JG94	Thioredoxin superfamily protein	0.0490381454288248
A0A5S9XM13	Phenylalanyl-tRNA synthetase	0.0475091862667336
A0A7G2FBV7	(thale cress) hypothetical protein	0.0470688581367441
P09468	ATP synthase epsilon chain, chloroplastic	0.0457867719032497
Q56YA5	Serine--glyoxylate aminotransferase	0.0455378229293713
A0A654FSL1	AMP-binding domain-containing protein	0.0439267926587978
P21240	Chaperonin 60 subunit beta 1, chloroplastic	0.0438790813209001
A0A178UAN3	Succinate dehydrogenase [ubiquinone] flavoprotein subunit, mitochondrial	0.043235322302434
A0A178ULV9	RPS1	0.04288315567143
A0A178W4Q5	Glyceraldehyde-3-phosphate dehydrogenase	0.0425445958399157
Q94K48	ARM repeat superfamily protein	0.0419039689837712
A0A7G2DW29	(thale cress) hypothetical protein	0.041767660839791
A0A178UE63	(thale cress) hypothetical protein	0.0415579318099845

Q8LDY5	40S ribosomal protein S2	0.0413372326907482
A0A2P2CLF9	ATP synthase subunit alpha	0.0397041547498232
A0A178WAY6	(thale cress) hypothetical protein	0.0395148505725938
A0A1P8AWY1	High chlorophyll fluorescence phenotype 173	0.0390186970019925
A0A654G449	Photosystem II stability/assembly factor	0.0383931648862551
Q0WN31	Phosphoglucomutase (alpha-D-glucose-1,6-bisphosphate-dependent)	0.0381922937337033
Q9SGT3	Obg-like ATPase 1	0.0377401311264958
A0A178V7Z2	ESM1	0.0375805981899633
A0A178VHJ5	Ketol-acid reductoisomerase	0.0374582305393425
Q9SUI9	ATP synthase gamma chain, chloroplast (Fragment)	0.0369565863259925
Q8LA13	DEAD-box ATP-dependent RNA helicase 11	0.0364107038077885
A0A178VES0	SEX4	0.0359863469280588
A0A654E907	ATP-dependent Clp protease proteolytic subunit	0.0349692515835434
A0A5S9XRW2	Uncharacterized protein	0.0348376923415428
A0A178WLU9	PMEAMT	0.0344083474405973
A0A7G2F1A7	(thale cress) hypothetical protein	0.0327605630300167
A0A1B1W4Y4	Photosystem I iron-sulfur center	0.0325210158132973
A0A7G2F3G0	(thale cress) hypothetical protein	0.0325023744281634
A0A654G2F8	5-methyltetrahydropteroyltriglutamate--homocysteine S-methyltransferase	0.0314615555504485
A0A075M4Z6	Ribulose bisphosphate carboxylase small subunit (Fragment)	0.03005903770089
A0A384LEC5	(thale cress) hypothetical protein	0.0298224035608309
O03042	Ribulose bisphosphate carboxylase large chain	0.0295316272332207
Q96292	Actin-2	0.0293071882129001
A0A1I9LPQ6	Glyoxylate reductase 1	0.0284661125726453
A0A654EW54	Elongation factor G, chloroplastic	0.0273731820210991
A0A5S9VCL0	Catalase	0.0267422181532828
Q5D6C5	Adenosylhomocysteinase	0.0265926872106019
O23553	Beta-amylase 3, chloroplastic	0.0252367176634214
A0A1P8BDK6	ADP-ribosylation factor A1B	0.0251154232949351
P38418	Lipoxygenase 2, chloroplastic	0.0246862766539092
A0A178V5M8	Phosphoglycerate kinase	0.0240727855332719
A0A1B1W4V2	ATP synthase subunit beta, chloroplastic	0.02336150269806
B9DI55	AT3G09840 protein	0.0232195551354692
A0A178WGH9	Vacuolar proton pump subunit B	0.0231364328173908
A0A178VB65	Clathrin heavy chain	0.0231107454896176
A0A178V1L3	(thale cress) hypothetical protein	0.021310319324002
A0A1I9LQ23	Cyclophilin-like peptidyl-prolyl cis-trans	0.0210448174344809

	isomerase family protein	
A0A178V993	CCT-theta	0.0203969456396582
A0A178UW98	Fructose-bisphosphate aldolase	0.020010825202915
A0A1B1W4X6	30S ribosomal protein S3, chloroplastic	0.0189241014233623
F4I0N7	GAST1 protein homolog 1	0.0187741713611948
Q94BT5	Glutathione transferase	0.0185328034501438
A0A654F988	S5 DRBM domain-containing protein	0.0172286501800425
Q56WK6	Patellin-1	0.0167833932257818
A0A178UYX1	Serine hydroxymethyltransferase	0.0166740988735061
A0A178WG36	(thale cress) hypothetical protein	0.016273473541384
Q9S726	Probable ribose-5-phosphate isomerase 3, chloroplastic	0.0159821347291761
A0A178WBS0	(thale cress) hypothetical protein	0.015862737576874
B9DG17	40S ribosomal protein SA	0.0151721782253371
A0A6B9JEL0	Small RNA binding protein 1	0.0137325043510122
O49485	D-3-phosphoglycerate dehydrogenase 1, chloroplastic	0.0135915135019921
A0A178WD57	Ribulose bisphosphate carboxylase small subunit	0.0118904077094144
A0A5S9WML4	Dihydrolipoyl dehydrogenase	0.0101967530596332
Q56X90	Carbonic anhydrase	0.0101393642044479
A0A5S9X6E0	RNA helicase	0.0100436960060098
Q9FLX7	Probable NADH dehydrogenase [ubiquinone] 1 alpha subcomplex subunit 5, mitochondrial	0.00954323092317385
A0A178URC5	S-(hydroxymethyl)glutathione dehydrogenase	0.00890219397589746
A0A178WC68	Eukaryotic translation initiation factor 5A	0.00874540457659338
A0A178W5L2	Tubulin alpha chain	0.0238311001147661
A1L4Y1	ATPase GET3B	
A0A178W3D8	(thale cress) hypothetical protein	
A0A654G681	Cysteinyl-tRNA synthetase	
A0A1P8BCV2	Acetyl-coenzyme A synthetase	
O04485	Leucyl-tRNA synthetase	
A0A654EWQ2	Uncharacterized protein	
A0A5S9WTE4	Uncharacterized protein	
F4IG09	Subtilisin-like protease SBT4.1	
F4J5M9	Clathrin light chain 3	
A0A7G2EIB7	Inositol hexakisphosphate and diphosphoinositol-pentakisphosphate kinase	
A0A5S9XAD8	HATPase_c domain-containing protein	
A0A178V0W7	Importin subunit alpha	
A0A7G2F1T1	(thale cress) hypothetical protein	
A0A7G2FCC7	(thale cress) hypothetical protein	
A0A178W962	D-3-phosphoglycerate dehydrogenase	

A0A654F0F3	Eukaryotic translation initiation factor 3 subunit F	
A0A1P8ARS7	CCT-epsilon	
B9DHI9	AT1G09640 protein	
A0A178W448	(thale cress) hypothetical protein	
A0A5S9Y814	Threonyl-tRNA synthetase	
Q8VYP7	Putative Sar1 GTP binding protein	
A0A178VYF3	Glutathione peroxidase	
A0A178VL71	(thale cress) hypothetical protein	
A0A5S9X8K6	Chromo domain-containing protein	
A0A178VC46	Uncharacterized protein	
O22914	Calvin cycle protein CP12-1, chloroplastic	
O23140	AP-2 complex subunit mu	
A0A178V430	(thale cress) hypothetical protein	
A0A654FNZ0	Uncharacterized protein	
A0A7G2F2G9	Sulfate adenylyltransferase	
A0A654FPF3	Ferredoxin	
B9DHM5	Pyruvate, phosphate dikinase	
A0A7G2F097	Ribosomal protein L15	
A0A178WA13	Proteasome subunit alpha type	
A0A7G2EY26	Proteasome subunit beta	
A0A178WCQ3	Proteasome subunit beta	
A0A654FYV3	MPN domain-containing protein	
Q56ZC8	Hydroxyacylglutathione hydrolase cytoplasmic (Fragment)	
A0A654F8W6	26S proteasome non-ATPase regulatory subunit 1 homolog	
A0A654F735	Pectinesterase	
A0A654FH19	Eukaryotic translation initiation factor 3 subunit C	
A0A654ENJ4	GDP-L-fucose synthase	
B9DHI2	AT4G21210 protein	
A0A178V235	(thale cress) hypothetical protein	
O49675	Probable carotenoid cleavage dioxygenase 4, chloroplastic	
Q2V4Q4	AT1G07320 protein	
A8MQM3	Ribose-phosphate diphosphokinase	
O65282	20 kDa chaperonin, chloroplastic	
A0A654GEW7	Uncharacterized protein	
A0A7G2DT19	(thale cress) hypothetical protein	
A0A7G2DT14	(thale cress) hypothetical protein	
O65572	Carotenoid 9,10(9,10)-cleavage dioxygenase 1	
A0A178V385	Fructose-bisphosphate aldolase	
A0A5S9XYN6	Uncharacterized protein	
A0A178WCC0	(thale cress) hypothetical protein	

A0A7G2EII3	(thale cress) hypothetical protein	
A0A654FCH5	Uncharacterized protein	
A0A7G2EHQ0	Phosphomannomutase	
A0A7G2EGL3	(thale cress) hypothetical protein	
C0Z300	AT2G37660 protein	
A0A1P8B070	Hydroxymethylglutaryl-CoA lyase	
A0A178VJ65	Proteasome subunit alpha type	
A0A654F8R2	Proteasome subunit alpha type	
A0A1P8B3B6	Adaptin family protein	
A0A178VXA5	(thale cress) hypothetical protein	
A0A178VWV6	(thale cress) hypothetical protein	
A0A654EW41	Succinate--CoA ligase [ADP-forming] subunit beta, mitochondrial	
A0A178WBP5	(thale cress) hypothetical protein	
Q1H5F3	At3g43810	
P11139	Tubulin alpha-1 chain	
A0A5S9WVV8	30S ribosomal protein S17, chloroplastic	
A0A178VH97	(thale cress) hypothetical protein	
A0A5S9XP07	S-adenosylmethionine synthase	
A0A7G2EUY8	Acetolactate synthase	
A0A654FHK1	Protein kinase domain-containing protein	
Q0WLB0	Xyloglucan endotransglucosylase/hydrolase	
A0A178VT47	Phosphopyruvate hydratase	
A0A654FGI2	D-fructose-1,6-bisphosphate 1-phosphohydrolase	
P25873	50S ribosomal protein L15, chloroplastic	
P27521	Chlorophyll a-b binding protein 4, chloroplastic	
A0A654FE88	CCT-alpha	
A0A178UXP7	Tubulin alpha chain	
P29517	Tubulin beta-9 chain	
Q0WL48	Uncharacterized protein At1g20440	
A0A654F7V9	TCTP domain-containing protein	
Q8H7B4	Glucan endo-1,3-beta-D-glucosidase (Fragment)	
Q5PNZ9	At1g22780	
A0A178UA91	(thale cress) hypothetical protein	
P37107	Signal recognition particle 54 kDa protein, chloroplastic	
A0A178U7L7	TGG1	
A0A5S9XFR2	SAICAR synthetase	
P38420	DNA-directed RNA polymerase II subunit 2	
A0A5S9XKB3	TRASH domain-containing protein	
A0A178UXQ6	Nucleoside diphosphate kinase	
P40940	ADP-ribosylation factor 3	
B9DGS0	ADP/ATP translocase	
A0A178UQ77	GTP-binding nuclear protein	

P42036	40S ribosomal protein S14-3	
A0A5S9XQK2	14_3_3 domain-containing protein	
A0A178UA42	(thale cress) hypothetical protein	
A0A178VJ06	(thale cress) hypothetical protein	
F4K015	Dynamin-like protein	
A0A178WAZ3	Plastocyanin	
A0A178UPD3	(thale cress) hypothetical protein	
A0A178V326	(thale cress) hypothetical protein	
F4JD01	Proteasome subunit beta	
A0A384KNV6	(thale cress) hypothetical protein	
A0A654GDN2	Glutamate-1-semialdehyde 2,1-aminomutase	
A0A178UA36	SRK2G	
A0A654FDY3	CN hydrolase domain-containing protein	
Q94BW0	At1g35160/T32G9_30	
F4JTH0	Aspartate aminotransferase	
A0A654FS26	Gamma-ECS	
A0A178V0A3	Glutathione transferase	
A0A7G2EY35	Cysteine synthase	
Q0WW95	Cysteine synthase	
P48348	14-3-3-like protein GF14 kappa	
A0A178UNB4	(thale cress) hypothetical protein	
A0A178VP20	(thale cress) hypothetical protein	
A0A178UJU5	(thale cress) hypothetical protein	
Q7G923	60S ribosomal protein L27A; 71521-71081	
F4KDU5	Ribosomal protein L4/L1 family	
F4I3L1	Phosphoglycerate kinase	
A0A178VF24	(thale cress) hypothetical protein	
A8MS28	Ribosomal L27e protein family	
A0A178VXY6	Glutathione peroxidase	
Q541W9	Uncharacterized protein At3g12110/T21B14_108	
A0A7G2F0R0	Protein-methionine-S-oxide reductase	
A0A7G2F961	(thale cress) hypothetical protein	
A0A7G2F639	Glucose-1-phosphate adenylyltransferase	
A0A514YJ21	ATP synthase subunit b, chloroplastic	
A0A178VY74	Photosystem I P700 chlorophyll a apoprotein A1	
P56767	Photosystem I P700 chlorophyll a apoprotein A2	
A0A7G2FNE9	Cytochrome f	
A0A1B1W4W5	ATP-dependent Clp protease proteolytic subunit	
P56773	Cytochrome b6	

A0A514YJW6	50S ribosomal protein L16, chloroplastic	
A0A1B1W4W8	50S ribosomal protein L20, chloroplastic	
A0A1B1W4X3	50S ribosomal protein L33, chloroplastic	
A0A1B1W4W9	30S ribosomal protein S18, chloroplastic	
A0A1B1W4X5	30S ribosomal protein S19, chloroplastic	
A0A178VVS2	(thale cress) hypothetical protein	
A0A1B1W4W0	Photosystem II reaction center protein L	
A0A178VIZ0	PIP1A	
Q6KGZ1	30S ribosomal protein S7, chloroplastic	
A0A178V984	(thale cress) hypothetical protein	
A0A654FZJ9	ATP synthase subunit beta	
A0A178V4V1	(thale cress) hypothetical protein	
A8MS49	Phototropin 2	
A7KNI1	NADH dehydrogenase subunit 7 (Fragment)	
B9DHX4	Malate dehydrogenase (Fragment)	
A0A7G2E3W0	(thale cress) hypothetical protein	
Q39209	RNA binding protein (Fragment)	
B9DFR9	AT2G45960 protein	
Q06738	Low-temperature-induced 78 kDa protein	
Q93ZB4	Phytoene dehydrogenase	
B0FV99	At1g04270 (Fragment)	
A0A178UH82	RD22	
A0A7G2DWN9	(thale cress) hypothetical protein	
A0A7G2E8R3	(thale cress) hypothetical protein	
A0A1P8AU99	Rhodanese homologue 2	
A0A654EHJ3	Serine/threonine-protein phosphatase 2A 55 kDa regulatory subunit B	
A0A7G2F8C1	(thale cress) hypothetical protein	
Q0WVZ3	Eukaryotic translation initiation factor 3 subunit I	
A0A178VM60	Zeta-carotene desaturase	
A0A178VIW5	PR 65	
A0A178UG43	BIP2	
A0A7G2FBG2	Ferritin	
A0A5S9YHP7	(thale cress) hypothetical protein	
A0A178VVN9	(thale cress) hypothetical protein	
A0A178VNK7	PSBTN	
A0A178WD95	SKP1-like protein	
A0A178UBS9	40S ribosomal protein S21	
Q41188	Cold shock protein 2	
A8MQR4	60S acidic ribosomal protein P0	
A0A178UZT1	40S ribosomal protein S3a	
F4JWM1	Ribosomal protein S5 domain 2-like superfamily protein	
Q42560	Aconitate hydratase 1	

A0A5S9XKR6	OMPdecase	
A0A7G2DWI3	Carbamoyl-phosphate synthase (glutamine-hydrolyzing)	
Q0WSS0	Uncharacterized protein At1g57860	
A0A5S9XKG3	(thale cress) hypothetical protein	
A0A654G692	Glutamine synthetase	
A0A654GBM6	Aldo_ket_red domain-containing protein	
Q0WLJ0	Peroxisomal-3-keto-acyl-CoA thiolase 1	
A0A7G2DXI6	Thiamine-phosphate pyrophosphorylase	
A0A654G5U7	RRM domain-containing protein	
A0A7G2E2R1	Proteasome subunit beta	
Q29PY2	At5g12110	
A0A5S9TIS9	ATP phosphoribosyltransferase	
Q8GWE0	Pentatricopeptide repeat-containing protein At4g16390, chloroplastic	
A0A5S9Y1A3	Rhodanese domain-containing protein	
A0A7G2F5W0	Dihydrolipoyllysine-residue succinyltransferase	
A0A178VXZ7	(thale cress) hypothetical protein	
A0A178VQL0	(thale cress) hypothetical protein	
A0A178V304	Coatomer subunit zeta	
A0A7G2DT04	Pyruvate dehydrogenase E1 component subunit alpha	
A0A5S9XWB6	Usp domain-containing protein	
B7T504	Putative ATP-dependent Clp protease proteolytic subunit (Fragment)	
F4JXE7	RNA-binding (RRM/RBD/RNP motifs) family protein	
F4K180	RNA helicase	
A0A178V3F1	Serine/threonine-protein phosphatase	
G1JSI6	At1g59359	
F6M3L4	Pyridoxine biosynthesis protein	
A0A178V785	ATP-dependent Clp protease proteolytic subunit	
A0A7G2EL76	(thale cress) hypothetical protein	
A0A178US36	40S ribosomal protein S24	
A0A178VE56	60S ribosomal protein L27	
A0A178USJ2	ACHT2	
Q8LD03	40S ribosomal protein S7-3	
A0A178V2B3	Proteasome subunit beta	
A0A7G2E784	(thale cress) hypothetical protein	
A0A7G2F3R2	Xyloglucan endotransglucosylase/hydrolase	
A0A178V6Q6	(thale cress) hypothetical protein	
A0A5S9XD62	Thioredoxin domain-containing protein	
A0A384KKR2	(thale cress) hypothetical protein	
A0A7G2E2S1	(thale cress) hypothetical protein	
A0A178U8I7	Era-type G domain-containing protein	
A0A178UB34	FUS11	

A0A178V1W8	(thale cress) hypothetical protein	
A0A178UCD3	40S ribosomal protein S8	
A0A654FP28	Uncharacterized protein	
B9DGK9	Coatomer subunit delta	
A0A654FRT4	STI1 domain-containing protein	
A0A1P8B108	N-acetyl-glutamate semialdehyde dehydrogenase	
A0A654EX31	Uncharacterized protein	
A0A178UVE7	(thale cress) hypothetical protein	
A0A178UKW4	(thale cress) hypothetical protein	
A0A178UL10	CCT-beta	
A0A178W6I0	SNF4	
A0A178UB52	Isocitrate dehydrogenase [NAD] subunit, mitochondrial	
A0A654ETK5	(thale cress) hypothetical protein	
A0A178VHG0	Glutaredoxin-dependent peroxiredoxin	
Q8L8N9	Spore coat protein-like protein	
A0A654FIP0	Galactinol--sucrose galactosyltransferase	
A0A7G2DQW8	(thale cress) hypothetical protein	
A0A654G0X9	(thale cress) hypothetical protein	
A0A7G2FM21	(thale cress) hypothetical protein	
A0A178UWP5	3-isopropylmalate dehydratase	
F4J1V2	DNAJ homologue 3	
A0A5S9U051	Uncharacterized protein	
A0A5S9XZ93	Uncharacterized protein	
F4JQD8	Translation initiation factor 3 protein	
B3H5Y8	Glycine cleavage system P protein	
A0A5S9YIS7	Uncharacterized protein	
A0A5S9Y5Z9	Cytochrome b-c1 complex subunit Rieske, mitochondrial	
F4HVW0	Proteasome component (PCI) domain protein	
A0A7G2ERG6	(thale cress) hypothetical protein	
A0A178W0Z6	ATP synthase subunit gamma	
Q96262	Plasma membrane-associated cation-binding protein 1	
A0A5S9XG25	(thale cress) hypothetical protein	
Q0WUH6	Catalase	
Q56YY6	Uncharacterized protein At1g20110 (Fragment)	
A0A178WLT0	Eukaryotic translation initiation factor 5A	
A0A7G2E348	(thale cress) hypothetical protein	
A0A178UIY8	Uncharacterized protein	
Q8LEA5	Thioredoxin-dependent peroxiredoxin	
Q9C5Z2	Eukaryotic translation initiation factor 3 subunit H	
A0A178WI45	(thale cress) hypothetical protein	

A0A5S9WU24	Uncharacterized protein	
Q9C865	SH3 domain-containing protein 1	
A0A654EQA9	Ribosomal_L6e_N domain-containing protein	
A0A178WG16	3-oxoacyl-[acyl-carrier-protein] synthase	
A0A178W4H7	(thale cress) hypothetical protein	
A0A654G6Z8	6-phosphogluconate dehydrogenase, decarboxylating	
A0A654GB24	Pyruvate decarboxylase	
Q8L7R7	AT4g37510/F6G17_160	
A0A178U6W0	BXL1	
A0A7G2FG07	(thale cress) hypothetical protein	
B9DH43	AT5G65220 protein	
A0A178UGQ5	NEET	
Q8LEF5	50S ribosomal protein L27	
A0A178UNS2	Small ubiquitin-related modifier	
A0A7G2FK60	Dihydrolipoyllysine-residue succinyltransferase	
Q9FLT0	Ribonuclease TUDOR 2	
A0A7G2FGK7	Glucosidase 2 subunit beta	
A0A7G2FHT9	(thale cress) hypothetical protein	
A0A178UMU4	(thale cress) hypothetical protein	
B0ZVS7	NHL3 (Fragment)	
Q67ZX8	40S ribosomal protein S19-like	
A0A7G2F8Y2	Ubiquitinyl hydrolase 1	
Q8GUG4	Uncharacterized protein	
A0A654G1P3	Peptide deformylase	
A0A178VK18	6-phosphogluconate dehydrogenase, decarboxylating	
Q9FZ76	60S ribosomal protein L6-1	
Q0WW72	60S ribosomal Protein L12-like	
A0A7G2EIJ8	tRNA (guanine(26)-N(2))-dimethyltransferase	
A0A7G2ELB8	(thale cress) hypothetical protein	
Q93ZK4	AT3g26420/F20C19_14	
A0A7G2ERE5	(thale cress) hypothetical protein	
Q9LK61	30S ribosomal protein S10, chloroplastic	
A0A5S9VBJ8	Small nuclear ribonucleoprotein Sm D3	
A0A178WBT3	EDA10	
A0A7G2DV36	(thale cress) hypothetical protein	
A0A178W322	Alkaline/neutral invertase	
Q9LQR8	26S proteasome non-ATPase regulatory subunit 3 homolog B	
Q9LS02	Allene oxide cyclase 2, chloroplastic	
A0A178UPY9	(thale cress) hypothetical protein	
A0A654F8F2	(thale cress) hypothetical protein	
A0A178VBY8	(thale cress) hypothetical protein	
A0A7G2EN38	D-3-phosphoglycerate dehydrogenase	

A0A5S9XDV3	Uncharacterized protein	
A0A654GAI3	Methionine S-methyltransferase	
A0A5S9XC85	(thale cress) hypothetical protein	
A0A5S9XES0	DUF2470 domain-containing protein	
A0A5S9XD98	T-complex protein 1 subunit delta	
A0A5S9YCP5	Uncharacterized protein	
A0A178U7Z1	Amylomaltase	
A0A178UG88	(thale cress) hypothetical protein	
A0A7G2ENW7	(thale cress) hypothetical protein	
A0A178UPS5	Aminotran_1_2 domain-containing protein	
B3H4M0	Post-illumination chlorophyll fluorescence increase	
A0A178UUQ0	(thale cress) hypothetical protein	
Q9LY74	2-methyl-6-phytyl-1,4-hydroquinone methyltransferase, chloroplastic	
A0A5S9Y0T5	GST N-terminal domain-containing protein	
A0A5S9Y2X6	60S ribosomal protein L36	
A0A654FY85	Assimilatory sulfite reductase (ferredoxin)	
A0A178V9A8	60S ribosomal protein L7a	
A0A178VF73	(thale cress) hypothetical protein	
A0A654FTY1	Ribosomal_L28e domain-containing protein	
Q9M0N1	Plant UBX domain-containing protein 10	
A0A178UTB9	Vesicle-fusing ATPase	
F4J0X9	Homocysteine methyltransferase 2	
A0A178VG46	40S ribosomal protein S21	
A0A178VA59	60S ribosomal protein L36	
A0A6B7JDQ1	Proliferating cell nuclear antigen	
A0A178VB48	40S ribosomal protein S7	
A0A654F3F3	Uncharacterized protein	
A0A7G2E0Q6	(thale cress) hypothetical protein	
A0A654EGJ2	Uncharacterized protein	
A0A654F5U8	Ferritin	
A0A654EQK1	Uncharacterized protein	
A0A654ETC9	Chlorophyll a-b binding protein, chloroplastic	
Q9S834	ATP-dependent Clp protease proteolytic subunit 5, chloroplastic	
Q8GXQ8	Putative inosine-5-monophosphate dehydrogenase	
A0A178WF69	Obg-like ATPase 1	
F4JN17	Ankyrin repeat-containing protein 2	
A0A5S9Y678	(thale cress) hypothetical protein	
A0A7G2E0M6	(thale cress) hypothetical protein	
Q9SE60	Methylenetetrahydrofolate reductase 1	
A0A7G2ELV3	(thale cress) hypothetical protein	
A0A7G2EH20	(thale cress) hypothetical protein	
Q9SFH9	Delta-aminolevulinic acid dehydratase 1, chloroplastic	
A0A7G2EF58	(thale cress) hypothetical protein	

A0A178WH62	RPN12a	
Q9SHI1	Translation initiation factor IF-2, chloroplastic	
A0A654EW86	(thale cress) hypothetical protein	
A0A5S9X4B0	Sucrose-phosphatase	
A0A7G2EHP4	Citrate synthase	
A0A7G2DVD0	(thale cress) hypothetical protein	
A0A654F8M7	Protein kinase domain-containing protein	
A8MRE8	Triosephosphate isomerase	
A0A654EVY1	(thale cress) hypothetical protein	
Q9SKU1	Clathrin light chain 1	
A0A654EU45	Isocitrate dehydrogenase [NADP]	
Q9SN73	Protein BUNDLE SHEATH DEFECTIVE 2, chloroplastic	
A0A654FEK4	Malate dehydrogenase	
A0A178VDJ4	UDP-glucuronate decarboxylase	
A0A178V7M0	(thale cress) hypothetical protein	
A0A178VCP4	(thale cress) hypothetical protein	
A0A654FGH8	Uncharacterized protein	
Q9SRZ6	Cytosolic isocitrate dehydrogenase [NADP]	
A0A1I9LMY7	40S ribosomal protein S24	
Q9SSA5	Peptidyl-prolyl cis-trans isomerase CYP38, chloroplastic	
A0A178V791	Vacuolar protein sorting-associated protein 29	
A0A7G2E075	(thale cress) hypothetical protein	
A0A178W0C4	Serine/threonine-protein phosphatase	
Q0WWC5	Uncharacterized protein At1g48350	
A0A178WJK9	ATPI4K ALPHA	
B9DF86	Lipoyl(octanoyl) transferase (Fragment)	
A0A7G2FEQ0	Cysteine synthase	
A0A178W5Y6	Chlorophyll a-b binding protein, chloroplastic	
A0A7G2EWN1	Protein translocase subunit SecA	
A0A654FU27	Elongation factor Ts, mitochondrial	
Q9XF88	Chlorophyll a-b binding protein CP29.2, chloroplastic	
A0A7G2E1Y9	(thale cress) hypothetical protein	
Q9XFS9	1-deoxy-D-xylulose 5-phosphate reductoisomerase, chloroplastic	
Q0WWD3	Vacuolar-type H+-ATPase (V-ATPase) subunit D	
A0A178W7K9	MBD10	
A0A178VQU8	(thale cress) hypothetical protein	
B9DFC0	AT4G08870 protein	
A0A654FLS1	Thioredoxin domain-containing protein	
A0A7G2EV07	(thale cress) hypothetical protein	
F4INA0	Nicotinate-nucleotide pyrophosphorylase [carboxylating]	
A0A7G2EBV3	UDP-glucuronate decarboxylase	
A0A7G2FEV2	(thale cress) hypothetical protein	

Q94K56	GrpE protein homolog	
Q8LD96	Nodulin-like protein	
A0A178UM83	Eukaryotic translation initiation factor 3 subunit L	
A0A1P8BCE3	Octicosapeptide/Phox/Bem1p family protein	
Q93XY1	At5g53620	
A0A178UAT5	Uncharacterized protein	
A0A178UCE0	(thale cress) hypothetical protein	
Q9FXM2	DC 1.2 homolog (Fragment)	
Q9FM97	Pyruvate kinase	
Q9FJZ7	AT5G66420 protein	
A0A7G2FAP9	(thale cress) hypothetical protein	
Q9FIS4	ATP-dependent Clp protease ATP-binding subunit	
A0A1P8BG25	Glycosyl hydrolase family protein	
Q9FM47	RNA-binding (RRM/RBD/RNP motifs) family protein	
A0A7G2FNH5	(thale cress) hypothetical protein	
Q93VS8	ARM repeat superfamily protein	
Q9LX13	(3R)-hydroxymyristoyl-[acyl-carrier-protein] dehydratase	
A0A178UT81	Carbonic anhydrase	
Q8L816	Uncharacterized protein At4g09520	
Q9SMQ9	DnaJ-like protein	
A0A178UWN8	N-acyl-L-amino-acid amidohydrolase	
A0A654FVA8	ENDO3c domain-containing protein	
Q9M0Y0	Uncharacterized protein AT4g05150	
F4JEJ3	Metallo-hydrolase/oxidoreductase superfamily protein	
Q9LX44	FBD-like domain family protein	
Q9SD66	Thylakoid soluble phosphoprotein	
Q42338	AT3G48140 protein	
A0A654FGL7	Eukaryotic translation initiation factor 3 subunit G	
Q9LF93	Pyruvate kinase	
A0A7G2EUV4	Small nuclear ribonucleoprotein Sm D2	
Q9SVD1	At3g52500	
Q9M1Q0	RING-type E3 ubiquitin transferase	
Q9SIJ8	Adenine nucleotide alpha hydrolases-like superfamily protein	
Q8LBA5	Putative monooxygenase	
Q9SIE3	(3R)-hydroxymyristoyl-[acyl-carrier-protein] dehydratase	
A0A7G2EDL4	(thale cress) hypothetical protein	
Q9CA53	Uncharacterized protein F1M20.32	
Q9SHG8	At1g17100	
Q9LPS0	F11F12.19 protein	
Q9C9G5	Adenine nucleotide alpha hydrolases-like superfamily protein	
Q9C685	At1g51100	

Q9C9I7	Rieske (2Fe-2S) domain-containing protein	
A0A654EJB7	DUF2431 domain-containing protein	
A0A178WBF4	DNL-type domain-containing protein	
Q9FZG2	T2E6.7	
Q9C7S3	Inner membrane localized protein	
Q9FE18	Peptidyl-prolyl cis-trans isomerase	
Q8VZ31	Tetratricopeptide repeat (TPR)-like superfamily protein	
A0A178WEL2	(thale cress) hypothetical protein	
A0A7G2DYR6	(thale cress) hypothetical protein	
A0A7G2E766	(thale cress) hypothetical protein	
Q9SSG7	F25A4.12 protein	
A0A5S9WTK4	ANAPC4_WD40 domain-containing protein	
Q0WUH5	Uncharacterized protein At1g19691	
Q680H0	mRNA, clone: RAFL22-54-C15	
B9DGR7	AT1G49750 protein (Fragment)	
A0A1I9LMI8	Transmembrane protein	
A0A1I9LQL1	GDSL-like lipase/acylhydrolase superfamily protein	
A0A1P8ART2	Molecular chaperone Hsp40/DnaJ family protein	
F4HSW8	GYF domain-containing protein	
Q9LPP9	F15H18.21	
Q8LAP5	Protein-synthesizing GTPase	
Q9FFP6	Pyruvate kinase	
Q94JV2	AT3g27050/MOJ10_14	
Q9LIK1	ADP-ribosylation factor C1	
Q9C6U3	Uncharacterized protein T8G24.2 (Fragment)	
Q93VK7	AT5g14910/F2G14_30	
O82762	F17H15.1/F17H15.1	
A0A7G2F273	(thale cress) hypothetical protein	
A0A5S9Y7L0	MADS-box domain-containing protein	
A8MQJ8	Glucose-induced degradation-like protein	
A0A654FCY2	Lipoxygenase	
F4JE18	Dentin sialophosphoprotein-like protein	
F4I3P5	Glutamine amidotransferase	
A0A7G2F5E9	Isocitrate dehydrogenase [NAD] subunit, mitochondrial	
Q9FVP6	3-phosphoshikimate 1-carboxyvinyltransferase	
Q9LMR1	At1g15730/F7H2_7	

Supplementary Table S4 The list of the primer sequences in this study.

Primers	sequences (5'to3')
**BiFC assay**	
IN-GRF6-F1 (pRTVc-Venus)	TATCCAGATCCAGTGGGATCCATGGCGGCGACATTAGG
IN-GRF6-R1 (pRTVc-Venus)	CGCACTAGTAAGCTTGGTACCCATAGAGTAGTAATAACTCAGCACACACG
IN-GRF8-F1 (pRTVc-Venus)	TATCCAGATCCAGTGGGATCCATGGCGACGACCTTAAGCA
IN-GRF8-R1 (pRTVc-Venus)	CGCACTAGTAAGCTTGGTACCATATGCGAGTTTCTGATGATGC
IN-TUBB9-F1 (pRTVc-Venus)	TATCCAGATCCAGTGGGATCCATGGTTGGATCTTTGGAATCTG
IN-TUBB9-R1 (pRTVc-Venus)	CGCACTAGTAAGCTTGGTACCCTTGTGAGCTGGACAGATGC
IN-Histone4-F1 (pRTVc-Venus)	TATCCAGATCCAGTGGGATCCATGTCAGGAAGAGGAAAAGGAG
IN-Histone4-R1 (pRTVc-Venus)	CGCACTAGTAAGCTTGGTACCACCACCAAATCCATATAGAGTTCTTC
IN-TUB3-F1 (pRTVc-Venus)	TATCCAGATCCAGTGGGATCCATGCGTGAGATTCTTCACATCC
IN-TUB3-R1 (pRTVc-Venus)	CGCACTAGTAAGCTTGGTACCGTACTCTTCTTCCTGTTGGTATTCAC
MORF2-F1(pRTVc-Venus)	TATCCAGATCCAGTGGGATCCATGGCTTTGCCTTTGTCTGG
MORF2-R1(pRTVc-Venus)	CGCACTAGTAAGCTTGGTACCTCTTGTGTTTTCTCTGCGGC
IN-VIPP1-F1 (pRTVc-Venus)	TATCCAGATCCAGTGGGATCCATGGCTCTCAAAGCTTCACCT
IN-VIPP1-R1 (pRTVc-Venus)	CGCACTAGTAAGCTTGGTACCAAAGTCGTTAGCTTTCCTTCGC
IN-TRX1-F1 (pRTVc-Venus)	TATCCAGATCCAGTGGGATCCATGGCTTCGGAAGAAGGAC
IN-TRX1-R1 (pRTVc-Venus)	CGCACTAGTAAGCTTGGTACCAGCCAAGTGTTTGGCAATG
IN-NOT3-F1 (pRTVc-Venus)	TATCCAGATCCAGTGGGATCCATGGGTGCGAGCCGG
IN-NOT3-R1 (pRTVc-Venus)	CGCACTAGTAAGCTTGGTACCTACGACGAGTTCATCTTCAAGATAAC
IN-PSRP3-F1 (pRTVc-Venus)	TATCCAGATCCAGTGGGATCCATGATGTCATCAATGGCACTCT

IN-PSRP3-R1 (pRTVc-Venus)	CGCACTAGTAAGCTTGGTACCTTGAGAAGTTAAATTGCCGC
IN-PSRP5-F1 (pRTVc-Venus)	TATCCAGATCCAGTGGGATCCATGGCTCTTCTCTGCTTCAATT
IN-PSRP5-R1 (pRTVc-Venus)	CGCACTAGTAAGCTTGGTACCGACATTCTTGTTTTTCTTCATCTTG
IN-WHY1-F1 (pRTVc-Venus)	TATCCAGATCCAGTGGGATCCATGTCGCAACTCTTATCGACT
IN-WHY1-R1 (pRTVc-Venus)	CGCACTAGTAAGCTTGGTACCTCTATTCCATTCATAGTCTCC
IN-WHY3-F1 (pRTVc-Venus)	TATCCAGATCCAGTGGGATCCATGTCGCAGCTGTTATCTTC
IN-WHY3-R1 (pRTVc-Venus)	CGCACTAGTAAGCTTGGTACCTCTACTCCATTCGTAGTCTCCT
WHY1-cut-R2-1(pRTVc-Venus)	CGCACTAGTAAGCTTGGTACCTGCAGAGATAAGAACAGCAAACTC
WHY1/3-cut-R3-1(pRTVc-Venus)	CGCACTAGTAAGCTTGGTACCCGAATGACCCACGTAAAATCTAG
WHY3-cut-R2-1(pRTVc-Venus)	CGCACTAGTAAGCTTGGTACCAGCAGAGATAAGAACAGCAAACTCT
WHY1-cut-F2-1(pRTVc-Venus)	TATCCAGATCCAGTGGGATCCATGATATACAAAGGGAAAGCTGCGT
WHY1-cut-F3-1(pRTVc-Venus)	TATCCAGATCCAGTGGGATCCATGTTCAATTTTGTCTTGCCTTACCT
WHY3-cut-F2-1(pRTVc-Venus)	TATCCAGATCCAGTGGGATCCATGATTTACAAAGGGAAAGCTGCTC
WHY3-cut-F3-1(pRTVc-Venus)	TATCCAGATCCAGTGGGATCCATGTTCAACTTCGTCTTGCCGC
UBQ-CX-F1	GGGTTTCATAGATATCATCCGA
Venus-N-R(CX)	GAACTTGTGGCCGTTTAC
Venus-N-R2 （CX） -HA	CGTAATCTGGAACATCGTATGG
Venus-C-R(CX)	GTCCTCGATGTTGTGGCGGA
**subclone**	
pnWHY1-p3301-F1（Infusion）	tgacctcgagactagtATGTCGCAACTCTTATCGACT
pnWHY1-p3301-R1（Infusion）	aggtggaggtcccccgggTCTATTCCATTCATAGTCTCC
pnWHY3-p3301-F	TGACCTCGAGACTAGTATGTCGCAGCTGTTATCTTC
pnWHY3-p3301-R	AGGTGGAGGTCCCCCGGGTCTACTCCATTCGTAGTCTCCT

PSRP3-F	ACAAAAAAGCAGGCTATGATGTCATCAATGGCACTCT
PSRP3-R	CAAGAAAGCTGGGTCTTATTGAGAAGTTAAATTGCCGC
PSRP5-F	ACAAAAAAGCAGGCTATGGCTCTTCTCTGCTTCAATT
PSRP5-R2	CAAGAAAGCTGGGTCTTAGACATTCTTGTTTTTCTTCATCTTG
GRF6-F	ACAAAAAAGCAGGCTATGGCTATGGCCACAAGG
GRF6-R	CAAGAAAGCTGGGTCTTAAGCAACAGGGAGAGAATCTTC
**qPCR**	
q ACTIN2-F	AAGCTCTCCTTTGTTGCTGTT
q ACTIN2-R	GACTTCTGGGCATCTGAATCT
q WHY1-F	TTTTACGTGGGTCATTCGAT
q WHY1-R	GTCCACTGTTAACGCAGCTT
q WHY3-F	ACGGTGAAGTCTCGACAAAG
q WHY3-R	ATCGAATGACCCACGTAAAA
q grf6-F	CAATCGACTCCACCGTCTTT
q grf6-R	TCCCATAAACCCTTGCATTCT
qPSRP3-F	GGTGGTGAAGCCAGATGAGAA
qPSRP3-R	AGTGGGATTGTGCCATGTCC
qPSRP5-F	CAACGCTGTCGAGTCCAAGA
qPSRP5-R	GGTTCCTCCTTGCTCTCAGG
**mutant screen**	
psrp3	FP: ATCTCAACCATCAAGCGTTTG
	RP: TGTGGCTGTCAAATGTCATTC
psrp5	FP: CAACGACGGAGATTCAAGAAG
	RP: AACGGAAACGCTCTCTTTCTC
grf6	FP: AAGATGCAGAATCCGAAATAATG
	RP: ACAACAACTCCAGCATCAACC
why1	FP: TGACCAACAAACTGTTGATGG
	RP: TCGAATGACCCACGTAAAATC
why3	FP: TTGAAAAACAACAATCCATCC
	RP: GCTTCCCATTTTTCAAAGCTC
